# Chemical Exchange Saturation Transfer Imaging in Neuroinflammation: Methods, Challenges, and Recommendations

**DOI:** 10.3390/ijms262211059

**Published:** 2025-11-15

**Authors:** Emmanuel A. Mensah, Abrar Faiyaz, Giovanni Schifitto, Md Nasir Uddin

**Affiliations:** 1 Department of Biomedical Engineering, University of Rochester, Rochester, NY 14627, USA; 2 Department of Neurology, University of Rochester, Rochester, NY 14642, USA; 3 Department of Imaging Sciences, University of Rochester, Rochester, NY 14642, USA; 4 Department of Electrical and Computer Engineering, University of Rochester, Rochester, NY 14627, USA

**Keywords:** neuroinflammation, brain, MRI, CEST, neuroimaging, molecular imaging

## Abstract

Chemical Exchange Saturation Transfer (CEST) imaging has emerged as a promising non-invasive molecular MRI technique for investigating neuroinflammation. It offers unique insights into metabolic and molecular alterations in the brain. This review presents a comprehensive overview of CEST principles, methodological developments, and translational applications in neuroinflammation. It covers the basic mechanisms, pulse sequence designs, readout strategies, and various CEST contrasts used to probe molecular changes associated with inflammation. Recent advancements in fast CEST imaging, including optimized undersampling strategies and accelerated reconstruction methods are discussed. Improvements in post-processing and quantification techniques are also highlighted. The growing role of artificial intelligence (AI) in CEST imaging for image reconstruction, artifact correction, and biomarker extraction, is examined. Preclinical and clinical studies show CEST’s potential to detect neuroinflammation across neurological disorders. The impact of high-field MRI on enhancing CEST sensitivity and specificity are also discussed. Despite notable progress, several challenges remain. These include sensitivity to field inhomogeneities, lack of acquisition standardization, and limited clinical validation. We outline current limitations, translational barriers, and provide recommendations for improving reproducibility, facilitating clinical adoption, and integrating AI-based approaches for robust molecular characterization. Overall, CEST imaging shows great potential as a non-invasive biomarker for neuroinflammation. It can deepen understanding of the molecular and metabolic mechanisms underlying neurological diseases, while addressing technical and translational challenges remains key for its broader clinical implementation.

## 1. Introduction

Neuroinflammation is an immunological response within the central nervous system (CNS) associated with the activation of glial cells, release of inflammatory mediators, and disruption of the blood–brain barrier (BBB) [1]. This response can be primary or secondary. Primary neuroinflammation arises from immune dysregulation and autoimmune processes originating within the CNS and is characteristic of disorders such as multiple sclerosis (MS). Secondary neuroinflammation occurs when nonimmune events such as injury, degeneration, or metabolic stress trigger downstream inflammatory responses. This injury-driven inflammation contributes to conditions including Alzheimer’s disease (AD), Parkinson’s disease (PD), stroke, traumatic brain injury (TBI), amyotrophic lateral sclerosis (ALS), and healthy aging [2]. Depending on the disease stage and context, neuroinflammation can exert both protective and detrimental effects. In acute conditions such as stroke, TBI, or infections, it facilitates tissue repair and pathogen clearance, whereas chronic neuroinflammation in disorders like AD, PD, or MS contributes to neuronal damage, synaptic dysfunction, and disease progression, ultimately leading to cognitive decline [2,3,4]. As such, targeting neuroinflammation is essential for early diagnosis, monitoring disease progression and evaluating therapeutic responses across these disorders.

Several blood-based biomarkers have been investigated to assess neuroinflammation. Glial Fibrillary Acidic Protein (GFAP) reflects astrocytic reactivity. Soluble Triggering Receptor Expressed on Myeloid cells 2 (sTREM2) indicates microglial activation. Chitinase-3-like protein-1 (CHI3L1, also known as YKL-40) is associated with both astroglial and microglial activation. Inflammatory cytokines such as interleukin-6 (IL-6) and tumor necrosis factor-alpha (TNF-α) are commonly assessed. Although collecting blood is minimally invasive, and easier than a magnetic resonance imaging (MRI), it still involves discomfort, procedural risks, and limited feasibility for frequent or long-term monitoring [1,5,6].

To overcome these limitations, noninvasive imaging modalities such as positron emission tomography (PET), single-photon emission computed tomography (SPECT), and MRI have been used to study neuroinflammation[2,7]. PET, particularly with translocator protein (TSPO) ligands, such as [^11^C]PK11195, visualizes microglial activation [8]. SPECT, using tracers like [^123^I] CLINDE provides complementary insights into glial activity, albeit with lower spatial resolution than PET or MRI [9].

MRI offers a versatile, radiation-free platform for detecting and monitoring neuroinflammation through multiple contrast mechanisms. Conventional MRI sequences, such as T_2_-weighted (T_2_w) imaging and fluid attenuated inversion recovery (FLAIR) detect edema, gliosis, and white matter (WM) lesions. Diffusion MRI (dMRI) techniques, such as diffusion tensor imaging (DTI) and neurite orientation dispersion and density imaging (NODDI), capture microstructural alterations related to cytotoxic edema and axonal injury while susceptibility-weighted imaging (SWI) and quantitative susceptibility mapping (QSM) highlight iron accumulation indicating microglia activation [10,11,12]. Dynamic contrast-enhanced (DCE) T_1_-weighted (T_1_w) MRI assesses BBB disruption, a hallmark of neuroinflammation. Magnetic resonance spectroscopy (MRS) measures metabolites like choline (Cho), myo-inositol (MI), and N-acetylaspartate (NAA), reflecting glial activation and neuronal integrity, respectively [13].

Despite their strengths, PET and SPECT have limitations. Both involve ionizing radiation restricting their use in longitudinal studies and vulnerable populations such as children and older adults [14]. High costs, limited radioligand availability, and insufficient specificity for different tissue damage types further hinder clinical adoption [15,16,17].

Conventional MRI techniques, while widely available, lack molecular specificity, detecting only downstream structural or microstructural changes secondary to neuroinflammation. MRS provides metabolic information but suffers from low spatial resolution and limited sensitivity and is limited to small regions of interest (ROIs), precluding whole-brain assessments. Increasing signal averages or using multi-voxel MRS can improve quality but prolong acquisition times and multi-voxel MRS is prone to motion artifacts [14,18]. These constraints limit their clinical use for molecular level assessments on neuroinflammation.

Chemical Exchange Saturation Transfer (CEST) imaging is a promising MRI technique that helps overcome the limitations of other imaging modalities. CEST exploits the chemical exchange between labile protons on endogenous or exogenous molecules and bulk water, enabling the detection of specific metabolites at high resolution without ionizing radiation [19]. By selectively saturating exchangeable protons (e.g., in -OH, -NH, or -NH_2_ groups), CEST measures changes in metabolite concentration and tissue microenvironment [20,21]. Unlike PET or SPECT, CEST does not require radioactive tracers, and is readily implementable on clinical MRI scanners, making it well-suited for routine and longitudinal studies. Its high spatial resolution and molecular sensitivity make it ideal for investigating neuroinflammation [14].

Several CEST targets are relevant to neuroinflammation across neurological disorders [22,23,24]. In AD, CEST detects MI associated with astrocyte activation. In MS, glutamate and glycosaminoglycan alterations reflect excitotoxicity and demyelination, respectively. In PD, CEST detects glutathione depletion linked to oxidative stress and neurodegeneration [25]. In stroke, amide proton changes indicate tissue acidosis and lactate accumulation, marking ischemic injury. These molecular targets illustrate CEST’s potential to non-invasively detect molecular changes associated with neuroinflammation across various neurological diseases.

This review provides a comprehensive overview of CEST imaging for diagnosing and monitoring various neuroinflammatory diseases. Current clinical and preclinical applications, methodological advances, limitations, and specific recommendations are outlined to guide future CEST development for neuroinflammatory studies.

## 2. CEST-Detectable Targets in Neuroinflammation

Neuroinflammation induces molecular changes in brain tissue that CEST imaging detects by capturing proton exchange between specific molecules and water. One key change is tissue acidosis, resulting from increased anaerobic metabolism and immune cell activity [22]. For instance, in ischemic stroke, this acidic shift, driven by metabolic stress, alters pH-sensitive CEST signals such as Amide Proton Transfer-weighted (APTw) imaging. Because amide proton transfer is base-catalyzed, lower pH slows the exchange rate and decreases the CEST signal [26]. However, APTw contrast is not determined by pH alone. The measured signal also depends on saturation parameters, tissue temperature, water T_1_, and amide proton concentration. As such, these factors need to be considered when interpreting APTw as a biomarker of neuroinflammation.

The BBB breakdown in MS and AD is another significant change, allowing immune cell infiltration into brain tissue, contributing to neuroinflammation [27]. This process increases extracellular proteins and alters intracellular protein dynamics, enhancing the APTw signal, which primarily arises from intracellular amide protons in mobile proteins and peptides. Similarly, in PD, abnormal protein accumulation (e.g., α-synuclein) and neuroinflammatory changes may amplify the APTw signal by increasing intracellular mobile amide protons [28]. Thus, CEST imaging can reveal elevated protein levels associated with neuroinflammatory pathology.

Moreover, neuroinflammation activates glial cells, increasing metabolic turnover, and altering neurotransmitters and metabolite levels. For instance, encephalitis affects amine protons in molecules like glutamate, which resonate around 3 ppm, reflecting changes in neurotransmitter metabolism [29]. Similarly, hydroxyl protons in glucose, glycogen, and lactate (~0.5–1.5 ppm) are influenced by glycolytic activity. Elevated levels of these metabolites enhance hydroxyl CEST signal and provide insights into energy metabolism during neuroinflammation [14].

Neuroinflammation in MS causes demyelination, reducing lipid and protein content in myelin. This alters aliphatic protons, contributing to the relayed Nuclear Overhauser Effect (rNOE) signal at around −3.5 ppm. These rNOE signals, arising from CH_3_ and CH_2_ groups in lipids, reflect membrane damage and tissue remodeling in demyelinated MS lesions [30]. In PD, while demyelination is less prominent, changes in lipid membranes due to neuronal loss can also influence rNOE signals, reflecting microstructural alterations. However, rNOE contrast is strongly influenced by magnetization transfer contrast (MTC) and $B_{0}$/$B_{1}$ inhomogeneity, especially at ≥3 T, requiring robust corrections for reliable quantification [31].

**Figure 1:**
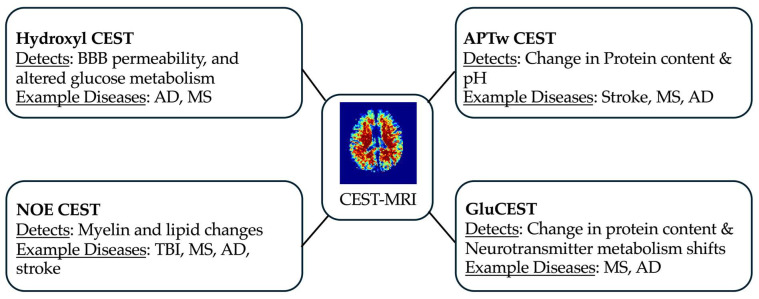
Applicability of CEST MRI for Neuroinflammation Studies. Different CEST contrasts (APTw, GluCEST, Hydroxyl, and NOE) reflect protein, neurotransmitter, osmolyte, and lipid changes, as well as the release of proinflammatory cytokines, enabling detection of neuroinflammatory processes across diseases such as stroke, multiple sclerosis (MS), Alzheimer’s disease (AD), and Traumatic brain injury (TBI).

By targeting proton pools like aliphatic, amide, and hydroxyl groups, CEST imaging detects molecular changes from tissue acidosis, protein accumulation, glial-driven metabolic changes, demyelination, and edema. This capability establishes CEST as a promising non-invasive tool for monitoring neuroinflammation and early disease diagnosis [32,33]. Figure 1 illustrates the potential application of various CEST MRI approaches for assessing neuroinflammation in neurological diseases.

## 3. CEST Imaging

### 3.1. Principles and Quantification

**Figure 2:**
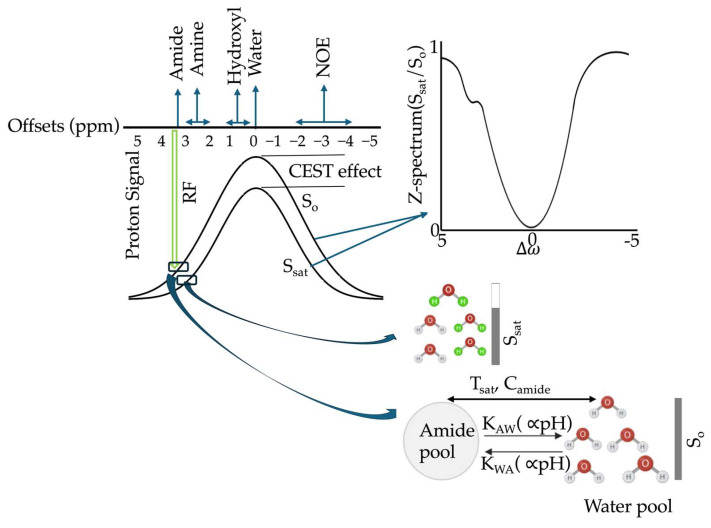
Schematic representation of the CEST mechanism. Exchangeable protons from different pools (amide, amine, hydroxyl, and aliphatic groups contributing to the nuclear Overhauser effect, rNOE) can be selectively saturated at specific frequency offsets relative to water. The saturation is transfered to the bulk water pool via chemical exchange, leading to a reduction in the water signal ($S_{\mathrm{sat}}$) compared to the unsaturated reference signal ($S_{0}$). Plotting the normalized water signal as a function of frequency offset yields the Z-spectrum, where dips correspond to exchangeable pools. The exchange rate between solute pools and bulk water is pH dependent, and the efficiency of proton transfer also depends on the saturation time ($T_{sat}$) and the concentration of amides ($C_{amide}$).

In the human body, water protons exist at high concentration (~111 M), whereas endogenous metabolites targeted by CEST MRI, such as amides, amines, and hydroxyl containing compounds, are present at much lower concentrations (μM to mM) [34]. This large concentration difference makes direct detection of metabolite signals challenging in standard MRI. CEST overcomes this limitation by applying frequency-selective radiofrequency (RF) saturation pulses at the resonance frequency of exchangeable protons in the solute pool, which is chemically shifted from the bulk water resonance. As these saturated protons exchange with bulk water protons, the saturation transfers to the water pool, cumulatively reducing its signal over time. Therefore, in CEST, saturation first reduces magnetization in the solute pool, and chemical exchange then transfers this saturation to the much larger water pool, producing a measurable reduction in water signal that indirectly reflects low-concentration metabolites. This indirect detection mechanism enhances sensitivity, enabling imaging of low-concentration compounds [19,20]. A schematic overview of the CEST mechanism is presented in Figure 2.

A typical CEST imaging sequence comprises three stages [35,36]. First, during the relaxation delay, longitudinal water magnetization recovers toward equilibrium. Second, during the saturation phase, a continuous-wave or pulsed RF preparation is applied at frequency offsets relative to the water resonance to selectively saturate exchangeable protons. Third, in the acquisition phase, the remaining water magnetization is flipped and spatially encoded, typically using a fast readout technique. This process is repeated across a range of saturation frequencies to generate the Z-spectrum, which represents the normalized water signal as a function of frequency offset. Therefore, the normalized water signal Z(Δω) is a function of offset frequency (Δω) and is defined as [19]:

$$Z(\Delta \omega )=\frac{S_{sat}(\Delta \omega )}{S_{0}}$$

where $S_{\mathrm{sat}\;}(\Delta \omega )$ is the signal after saturation at offset Δω, and $S_{0}$ is the unsaturated signal collected at a far resonant frequency from water.

The resulting CEST contrast is commonly quantified by the Proton Transfer Ratio (PTR), which is defined analytically for a two-pool system as [34,37]:

$$PTR=\frac{S_{0w}-S_{w}\left(t_{sat},\alpha \right)}{S_{0w}}=\frac{k_{sw}\cdot \alpha \cdot x_{CA}}{R_{1w}+k_{sw}\cdot x_{CA}}\left[1-e^{-\left(R_{1w}+k_{sw}\cdot x_{CA}\right)t_{sat}}\right]$$

where $S_{0w}$ is the equilibrium water signal, $S_{w}\left(t_{\mathrm{sat}},\;\alpha \right)$ is the signal after saturation, $k_{sw}$ is the exchange rate from solute pool to water pool, $x_{CA}$ is the fractional concentration of exchangeable solute protons, $t_{\mathrm{sat}\;}$ is the saturation time, and α is the labeling efficiency. The labeling efficiency is given by:

$$\alpha =\frac{\omega_{1}^{2}}{\omega_{1}^{2}+pq}$$

where

$$p=R_{2s}+k_{sw}-\frac{k_{sw}^{2}\cdot x_{CA}}{R_{2w}+k_{sw}\cdot x_{CA}},\;q=R_{1s}+k_{sw}-\frac{k_{sw}^{2}\cdot x_{CA}}{R_{1w}+k_{sw}\cdot x_{CA}}$$

CEST signal generation depends on several parameters. The exchange rate ($k_{sw})$ should be in the slow-to-intermediate range for selective saturation [26]; if it is too fast, the solute peak broadens, and saturation transfer becomes inefficient. Metabolite concentration determines the number of exchangeable protons, with higher concentration yielding stronger CEST signals. The saturation power $\left(B_{1}\right)$ and saturation time$\;(t_{\mathrm{sat}})$ determine how much magnetization is transferred [35,38]. Low $B_{1}$ and $t_{\mathrm{sat}}$ values yield weak labeling, while high values increase direct water saturation. Relaxation properties also play a role—longer $T_{1}$ supports greater signal accumulation while short $T_{2}$ leads to reduced signal due to rapid dephasing [39]. Finally, the frequency offset between the solute and water must be wide enough to minimize contamination from direct water saturation [26].

For CEST quantification, another widely used metric is the magnetization transfer ratio asymmetry ($MTR_{asym}$), which is conceptually similar to the PTR. It assumes sources such as direct water saturation (DS) and MTC are symmetric around the water resonance. Under this assumption, the total normalized water signal at a given offset can be expressed as [37]:

$$\frac{S_{w}(\Delta \omega )}{S_{0w}}=PTR(\Delta \omega )+MTC(\Delta \omega )+DS(\Delta \omega )$$

Since MTC and DS are assumed to be symmetric around water, MTR_asym_ quantifies the asymmetric CEST component as:

$$MTR_{asym}=\frac{S_{sat}(-\Delta \omega )-S_{sat}(\Delta \omega )}{S_{0w}}$$

Nevertheless, this symmetry assumption is often violated in CEST imaging, causing $MTR_{asym}$ to be contaminated by DS, MTC, and rNOE contributions. This limits its specificity to intended CEST effect at a particular offset (e.g., APTw signal at 3.5 ppm) [40]. To address this, APT# and NOE# metrics are derived using the extrapolated magnetization transfer reference (EMR) method. This approach fits Z-spectrum data at large offsets to model the semi-solid MTC background ($Z_{\mathrm{EMR}}$), enabling cleaner extraction of exchange effects [40,41]:

$$APT\#=Z_{EMR}\left(+3.5\;ppm\right)-Z\left(+3.5\;ppm\right)$$

$$NOE\#=Z_{EMR}\left(-3.5\;ppm\right)-Z\left(-3.5\;ppm\right)$$

EMR based metrics should be preferred when MTC asymmetry is a dominant confound, because they remove these contributions and improve specificity to amide and NOE pools. APT# is useful for APTw signal analysis, while NOE# aids in assessing lipid or protein environments in inflammatory conditions.

An additional metric used to assess CEST effect is the MTR with respect to exchange-dependent relaxation ($MTR_{Rex}$) which is defined as [31]:

$$MTR_{Rex}(\Delta \omega )=\frac{1}{Z_{lab}}-\frac{1}{Z_{ref}}$$

where $Z_{\mathrm{lab}\;}$ and $Z_{\mathrm{ref}\;}$ are the normalized water signal intensities at the labile and reference frequency offsets, respectively. $MTR_{Rex}$ also isolates exchange contributions by removing symmetric non-specific effects. Apparent Exchange-dependent Relaxation (AREX) metric is also used to compensate for longitudinal relaxation time $T_{1w}$, and is defined as [31]:

$$AREX(\Delta \omega )=\frac{MTR_{Rex}(\Delta \omega )}{T_{1w}}$$

AREX removes T_1_-driven biases and is particularly useful in pathologies with heterogeneous $T_{1}$ values such as MS and AD.

Another method is Lorentzian Difference (LD) map, which subtracts a fitted Lorentzian baseline from the experimental Z-spectrum [42]:

$$LD(\Delta \omega )=Z_{CEST}^{LF}-Z_{CEST}^{exp}$$

where $Z_{CEST}^{LF}$ is the Lorentzian fit and $Z_{CEST}^{exp}$ is the experimental Z-spectrum. It enhances sensitivity to CEST effects by suppressing symmetric background signals.

Magnetic Resonance Fingerprinting (MRF) has also been integrated with CEST to enable rapid quantitative mapping. In this approach, pseudorandomized saturation schedules generate unique signal trajectories that are matched to a Bloch-McConnell simulated dictionary to assign voxel wise parameters such as pool fraction and exchange rate. This approach provides fully quantitative maps, removes the need for full Z-spectra, reduces sensitivity to $B_{0}$ variation, and accelerates reconstruction [43,44].

Different MTC lineshapes are used to model background effects and improve separation of exchange contributions [40,45]. Lorentzian models suit liquid environments with symmetric and narrow profiles. Gaussian models represent intermediate broadening. Super-Lorentzian models describe semisolid tissues by capturing broad and restricted macromolecular motion and can be a reasonable assumption for neuroinflammatory studies. Pseudo-Voigt profiles, defined as a weighted sum of Gaussian and Lorentzian terms, have also been used as an effective approximation to a Voigt function [46]. Accurate multi-pool CEST quantification has further been demonstrated using spinlock-model-based fitting models [47]. Using an incorrect MTC lineshape can distort the estimated MTC amplitude, lead to improper spillover correction, bias APTw and NOE metrics, and ultimately confound the interpretation of metabolic or pH-related changes.

### 3.2. CEST Acquisition

CEST acquisition can be broadly divided into three phases [36,48]: recovery, CEST preparation, and readout. The recovery phase allows longitudinal magnetization to recover toward equilibrium before the CEST preparation. During the CEST preparation phase, selective RF saturation pulses are applied at specific frequency offsets to saturate exchangeable protons, transferring this saturation to bulk water through chemical exchange. Saturation schemes that balance efficiency and specific absorption rate (SAR) constraints are usually preferred. Common CEST saturation schemes include continuous-wave (CW), pulse-train (pCW), pulsed steady-state, and unevenly segmented or hybrid RF saturation schemes [49]. CW offers high efficiency but often exceeds SAR limits. pCW provides a good balance in SAR and sensitivity, making it the most used clinically. Pulsed steady state offers sharper images but have lower signal to noise ratio (SNR) compared to pCW, while hybrid schemes are more complex to model.

**Figure 3:**
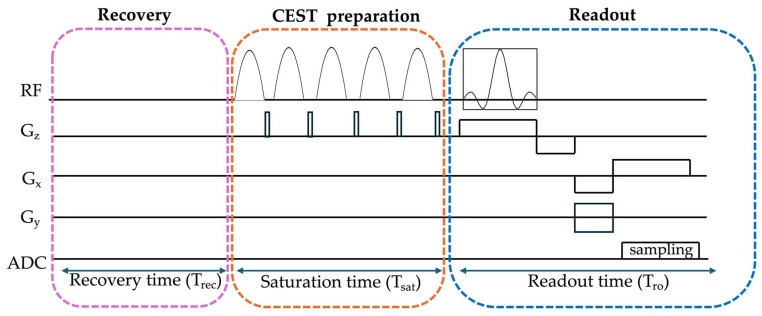
Sequence diagram for CEST imaging. The sequence includes relaxation recovery, CEST preparation module, and the readout. The preparation module consists of a train of Gaussian RF saturation pulses applied at a specific frequency offset, while the readout is a conventional GRE with sinc RF excitation pulse. Although GRE is shown, the CEST preparation module can be combined with different readout schemes depending on the application.

The readout phase follows, where rapid acquisition of the saturated signal is preferred to minimize relaxation effects and motion artifacts [49]. Consequently, various readout sequences have been developed to accelerate CEST imaging, including turbo spin echo (TSE), echo planar imaging (EPI), gradient echo (GRE) based sequences [36]. Figure 3 shows a schematic of a typical CEST sequence comprising the recovery, preparation, and readout phases.

### 3.3. Types of CEST Imaging

CEST imaging techniques are typically grouped into two categories, depending on the nature of the agents involved: paramagnetic (ParaCEST) and diamagnetic (DiaCEST) [50,51]. Each category differs in how it generates contrast and what molecular features it targets.

ParaCEST uses paramagnetic lanthanide (III) complexes with mobile protons on coordinated water molecules or on the ligand structure itself [51,52]. DOTA-based ligands chelate the lanthanide ion, producing a substantial shift in proton resonance frequency away from water and slowing exchange rate into a detectable range. SupraCEST, a subclass, enhances sensitivity by attaching the lanthanide complex to macromolecules with additional exchangeable protons, which further increases the chemical shift separation and optimizes the exchange rate [53]. ParaCEST agents are administered exogenously as contrast agents.

DiaCEST agents are exogenous or endogenous compounds that lack metal centers and generate contrast through chemical exchange of protons with water, typically without the large frequency shifts associated with paramagnetic agents [20]. They range from low-molecular-weight metabolites to macromolecules. Examples include ammonium chloride and iopamidol and iobitridol, used in AcidoCEST to map extracellular pH [54,55]. AcidoCEST uses the ratiometric analysis of CEST signals at ~4.2 and 5.6 ppm to provide concentration-independent, quantitative pH imaging, particularly useful for detecting tissue acidosis associated with neuroinflammatory processes [54]. Other agents include cationic polymers such as poly-L-lysine, which offer high proton density and improved sensitivity [56]. Thus, DiaCEST agents achieve molecular specificity by exploiting the exchange behavior of functional groups like amide and hydroxyl protons, without requiring paramagnetic components.

Endogenous CEST (EndoCEST), a subset of DiaCEST, exploits the body’s own metabolites and macromolecules as natural contrast sources [20]. It utilizes native molecules with exchangeable protons that can be detected in vivo without the need for exogenous injections. Common substrates include proteins and peptides (amide protons), amino acids and neurotransmitters (amine protons), and metabolites such as sugars and polyols (hydroxyl protons) [24]. These endogenous agents allow non-invasive mapping of biochemical parameters such as pH, metabolite concentration, and temperature. Here we discuss the various types of EndoCEST techniques and what they detect with regard to neuroinflammation.

#### 3.3.1. Amide Proton Transfer-Weighted (APTw)

APTw imaging is one of the most established endogenous CEST techniques. It detects the amide protons of mobile proteins and peptides, which resonate at ~3.5 ppm. The exchange of these amide protons typically occurs within the slow-to-intermediate regime, enabling effective CEST contrast at clinically available field strengths such as 3 T, with even greater sensitivity at higher fields [22,24]. APTw is sensitive to both tissue protein content and microenvironmental pH, as the exchange rate of amide protons is strongly pH dependent. Thus, it reflects proteomic density as well as tissue acidosis. In neuroinflammatory diseases, these properties are particularly relevant, as regions of inflammation may exhibit mild acidosis and altered protein turnover, leading to measurable changes in APTw signal [57]. Clinically, APTw has demonstrated utility in detecting ischemic injury through early pH alterations and in differentiating tumors and other lesions based on protein content. Overall, APTw provides contrast that maps protein concentration and pH abnormalities in the brain’s microenvironment, serving as a sensitive marker of tissue integrity and neuroinflammatory status.

#### 3.3.2. Glutamate-Weighted CEST (GluCEST)

GluCEST imaging is an MRI technique that detects the amine(-NH_2_) protons of glutamate, enabling non-invasive mapping of its concentration in the brain [58,59]. Glutamate is a key excitatory neurotransmitter, and its levels are tightly regulated under normal conditions. In neuroinflammatory diseases, GluCEST detects elevated glutamate caused by impaired synaptic clearance, glial activation, and BBB leakage, which causes extracellular glutamate accumulation, contributing to excitotoxic neuronal injury [29]. GluCEST signal increases in regions where immune activation enhances glutamate release or reduces uptake, such as in MS or autoimmune encephalitis. It reflects ongoing metabolic disturbances and has potential for identifying active lesions, tracking disease progression, and evaluating responses to therapies targeting glutamatergic or inflammatory pathways. GluCEST serves as a metabolic biomarker of glutamate dysregulation associated with neuroinflammation.

#### 3.3.3. Hydroxyl Proton CEST

Hydroxyl CEST imaging targets the exchangeable -OH protons (0.5–1.5 ppm from water) in endogenous molecules, with subtypes: glycosaminoglycan CEST (GagCEST), glycogen CEST (GlycoCEST), and glucose CEST (GlucoCEST) [59,60,61].

GagCEST quantifies glycosaminoglycans (GAGs) content in neural tissue, particularly in myelin and extracellular matrix structures. Neuroinflammation, as seen in MS, causes demyelination and degradation of GAG-rich matrices, resulting in reduced GagCEST signal in lesions. GlycoCEST captures glycogen and glycoprotein alterations; activated glial cells modify glycosylation patterns during inflammation, leading to detectable shifts in hydroxyl proton exchange. This makes GlycoCEST sensitive to inflammation-driven structural remodeling. GlucoCEST detects free glucose dynamics and is sensitive to immune cell metabolism. During neuroinflammatory responses, microglia and astrocytes upregulate glycolysis, increasing glucose uptake and -OH exchange, resulting in elevated GlucoCEST signals in lesions caused by encephalitis, AD-related neuroinflammation, or sepsis-associated encephalopathy. As such, hydroxyl proton CEST maps metabolic changes in neuroinflammation by exploiting the exchange characteristics of hydroxyl-bearing molecules altered during immune activation.

#### 3.3.4. Creatine CEST (CrCEST)

CrCEST imaging probes the guanidyl amine protons of creatine (Cr) and phosphocreatine (pCr), which resonate near 2 ppm from water [62]. These metabolites play a central role in cellular energy metabolism by buffering adenosine triphosphate (ATP) through the creatine kinase system. CrCEST enables high-resolution mapping of creatine distribution, offering a robust assessment of bioenergetic status with greater spatial specificity than MR spectroscopy [63].

In neuroinflammatory conditions, activated microglia and astrocytes exhibit altered energy demands, mitochondrial dysfunction and reduced creatine kinase activity [64]. This leads to diminished phosphocreatine turnover and lower CrCEST signal. This signal reduction reflects impaired ATP buffering and has been associated with lesion severity and progression in diseases such as MS [65]. As such, CrCEST provides a promising biomarker for evaluating metabolic dysfunction within inflamed neural tissue.

#### 3.3.5. Bacterial CEST (BacCEST)

BacCEST detects endogenous contrast arising from exchangeable protons in bacterial macromolecules, including proteins, carbohydrates, and metabolic byproducts [20]. These protons resonate within 1–4 ppm of water with signal intensity scaling with bacterial cell density [66]. Because it does not require exogenous agents, BacCEST enables non-invasive detection of bacterial presence based on intrinsic molecular signatures. Importantly, it can distinguish infectious neuroinflammatory processes from sterile inflammation. In bacterial encephalitis or cerebral abscesses, it detects elevated signals that correspond to the bacterial load, providing molecular specificity beyond conventional MRI [66,67]. This enables discrimination between infections and other inflammatory or neoplastic lesions and supports monitoring therapeutic response by quantifying bacterial reduction over time. Therefore, BacCEST provides imaging a biomarker for pathogen-driven neuroinflammation, supporting early diagnosis and guiding antibiotic interventions.

#### 3.3.6. Cryptococcus CEST (CryptoCEST)

CryptoCEST exploits the metabolic signature of *Cryptococcus* by detecting trehalose, whose hydroxyl protons (0.2–2 ppm, with a peak ~0.7 ppm), produce a distinct and quantifiable CEST signal [20,68]. In neuroinflammation, CryptoCEST is particularly suited for detecting cryptococcal meningitis, a severe opportunistic infection that can occur in HIV-infected individuals with low CD4 count and in other immunocompromised contexts [69]. Therefore, CryptoCEST provides a non-invasive means to assess fungal burden and track therapeutic response [20]. Its clinical utility lies in eliminating the need for repeated lumbar punctures and also offers high spatial specificity for lesion mapping.

#### 3.3.7. Nuclear Overhauser Enhancement (NOE) Imaging

NOE imaging is a magnetization transfer technique that detects dipolar cross-relaxation between non-exchangeable aliphatic protons (-CH_2_ and -CH_3_) in lipids and proteins and water protons [59,70]. These interactions produce negative signal changes up field from water, typically between −1.6 and −4 ppm, with a peak near −3.5 ppm. A major contributor to this signal is rNOE, where saturation of aliphatic protons is first transferred to nearby exchangeable protons and then relayed to water via chemical exchange [71]. Unlike CEST, which relies on chemical exchange, NOE reflects the spatial proximity and dipolar coupling between macromolecular and water protons, providing insight into lipid and protein microenvironments.

In neuroinflammatory diseases, NOE imaging is useful due to its sensitivity to changes in myelin and lipid content [30]. NOE has demonstrated utility in differentiating active from chronic plaques and tracking remyelination. It is also sensitive to lipid metabolism alterations associated with glial activation and oxidative stress, making it applicable to broader inflammatory diseases such as Alzheimer’s disease and autoimmune encephalitis [72].

### 3.4. Accelerating CEST MRI Acquisition

CEST imaging is usually long due to the need to acquire images at multiple frequency offsets to construct a Z-spectrum. This often limits its adoption in clinical settings for studying neuroinflammation and other applications. However, significant advances in pulse sequences, optimized k-space sampling and ordering, and image reconstruction techniques have been developed to accelerate CEST imaging.

EPI readouts were among the first used to achieve rapid CEST image acquisition. Zhou et al. demonstrated pH and APTw CEST contrasts in rats using spin-echo EPI [73,74]. This approach has further been improved through snapshot 3D EPI techniques, enabling whole-brain CEST [75]. However, EPI is highly sensitive to field inhomogeneity, often causing image distortions and lipid ghosting artifacts that can contaminate CEST signals [49]. Fat-suppression methods are typically needed to mitigate these artifacts.

To the best of our knowledge, TSE readouts are widely used for CEST, especially in clinical 3 T brain exams, as they offer robust image quality with minimal distortion or ghosting, even though they are relatively slow. Human APTw studies at 3 T used 3D TSE to visualize brain tumors [76]. The high flip-angle refocusing in TSE provides high SNR and artifact resilience, but at the cost of longer scan times and higher SAR [49]. This can limit 3D coverage or ultra-high-field applications.

GRE sequences enable fast CEST imaging with low SAR and flexible 3D acquisition, particularly at high fields. Dixon et al. and Zaiss et al. demonstrated rapid multi-slice and snapshot-CEST with GRE, respectively, with the latter achieving up to 16 slices in 7 s per offset [36,77]. Sedykh et al. further enhanced the GRE-based snapshot-CEST to achieve whole-brain APTw CEST in under 2 min using compressed sensing (CS) [78]. GRE offers faster readout with minimal distortion but suffers from lower SNR and sensitivity to $B_{0}$ inhomogeneity, limiting accuracy in regions with strong field variations.

Hybrid and advanced sequences, such as gradient and spin-echo (GRASE), sampling perfection with application-optimized contrasts using different flip angle evolution (SPACE), and balanced steady-state free precession (bSSFP), have been developed for faster CEST imaging [48,49].

GRASE combines TSE stability with the speed of EPI for CEST imaging. Zhu et al. demonstrated 3D GRASE CEST for whole-brain imaging in less than 10 min, prioritizing central k-space to minimize contrast loss. However, some artifacts remained due to $B_{1}$ field inhomogeneity and lipid signal contamination [79]. SPACE, an optimized 3D TSE with variable flip angles, achieves short echo spacing and long echo trains [49]. Zhang et al. implemented SPACE CEST for whole-brain APTw imaging in ~5 min with 2.7 mm isotropic resolution and 91% duty cycle, showing minimal susceptibility artifacts but requiring careful SAR management at high field strengths [80].

Balanced SSFP (True FISP), also known as bSSFP, was recently optimized for CEST imaging by Wu et al., who implemented a 3D single-shot readout at 3 T using a centric spiral reordering strategy [81]. Compared to FLASH-based CEST, the True FISP sequence demonstrated a significantly higher SNR in both phantom and in vivo experiments. Specifically, in an egg white phantom, APTw and NOE SNRs were increased by 68.3% and 57.0%, respectively. In healthy volunteers, temporal SNR at 3.5 ppm improved by 84%. These findings confirm True FISP’s suitability for rapid, high-quality 3D CEST readout in clinical settings. However, it remains sensitive to $B_{0}$ inhomogeneity and produces banding artifacts that can degrade image quality and affect CEST quantification. These effects become more pronounced at high magnetic field strengths [82].

#### 3.4.1. Optimizing Under-Sampling and k-Space Ordering for CEST Imaging

Fast CEST imaging is achieved through optimized k-space sampling and reordering techniques that undersample data while preserving CEST contrast. Maintaining contrast is crucial in CEST imaging, as the center of k-space carries most of the contrast information. As such, various k-space sampling methods and acquisition ordering strategies have been developed and demonstrated to enhance the efficiency of CEST imaging without compromising its contrast.

Conventional variable density sampling prioritizes dense central k-space acquisition for contrast preservation while sparsely sampling outer regions for acceleration. Heo et al. applied this approach to CEST, achieving an acceleration factor up to 4 [83]. Another method involves adapting volumetric interpolated breath-hold examination (VIBE) sequence to achieve a Snapshot CEST sequence capable of whole-brain coverage for APTw imaging with an acceleration factor 8.66 [78].

An additional sampling approach optimized for CEST imaging is frequency-offset-dependent (FOD) sampling, introduced by Liu et al. [42]. FOD uses an adaptive sampling mask for each offset that fully samples the central k-space universally but varies peripheral sampling probabilistically, allocating more samples toward the center of k-space for informative offsets like ±3.5 ppm, while applying sparser sampling at offsets farther from these frequencies. This enhances detection of CEST effects and enables whole-brain imaging at acceleration factors of up to 14, with minimal loss of CEST effect.

In addition, reducing the number of frequency offsets, focusing on those essential for contrast can also help minimize acquisition time. Sedykh et al. demonstrated that as few as seven offsets sampled around ±3.5 ppm was sufficient to retain APTw contrast in snapshot CEST [78]. Cheema et al. used Fisher information to identify the most informative offsets and trained a U-NET to reconstruct full Z-spectra from as few as 15 of 53 offsets, reducing scan time by a factor 3.5 [84]. Kwiatkowski et al. achieved 3 times faster CEST by using Z-spectrum sparsity with k-space and Z-spectrum principal component analysis (k-Z PCA) reconstruction, preserving spectral quality and MTR asymmetry accuracy [85]. Similarly, Bhattarai et al. employed an optimization algorithm and deep learning to reconstruct dense Z-spectra from just 10% offset points in mouse brains [86].

In terms of k-space ordering, centric phase-encoding is commonly preferred, as it acquires the contrast-rich center of k-space immediately after saturation. Spiral-centric ordering, used in snapshot-CEST by Zaiss et al., further improves acquisition efficiency while preserving saturation effects [36]. Non-Cartesian sampling trajectories including radial, periodically rotated overlapping parallel lines with enhanced reconstruction (PROPELLER), and rosette have been used for CEST imaging [87,88,89]. Radial acquisitions, demonstrated for whole-brain amide CEST mapping by Sui et al., improve motion tolerance by oversampling the k-space center, although they increase scan/reconstruction time and introduce a higher risk of aliasing [89]. Mahmud et al. applied rosette trajectories for fast CEST and MTC imaging using CS and achieved a scan time of 5.1 s per frequency offset, which was shown to be faster than other radial and Cartesian trajectories [87]. Rosette readouts provide incoherent k-space coverage and motion robustness and are particularly advantageous because $B_{0}$ maps can be estimated directly from rosette CEST data. However, they require complex sequence design and careful parameter tuning.

#### 3.4.2. Reconstruction

Reconstruction techniques complement CEST undersampling by enabling recovery of incomplete or noisy data. Among these, parallel imaging and CS represent two main acceleration strategies and are increasingly combined for improved efficiency. Parallel imaging, which utilizes multi-channel coils, operates either in image space using explicit coil sensitivity maps such as Sensitivity Encoding (SENSE) or in k-space by exploiting inter-coil redundancy as in GRAPPA [90,91]. In contrast, CS leverages sparsity in some domains of the signal (e.g., wavelet or total variation) to reconstruct from incoherently undersampled k-space [92].

Heo et al. combined CS with SENSE for CEST imaging, achieving 4-fold acceleration [83]. She et al. retrospectively demonstrated that parallel blind compressed sensing (PBCS), which integrates coil sensitivities with dictionary learning, can achieve a 10-fold acceleration for brain CEST imaging [93]. Their approach takes advantage of spatial-temporal redundancy in CEST data. Wada et al. applied 3D CS-SENSE CEST for glioma grading, enabling multi-slice APTw imaging with equivalent diagnostic performance to 2D, effectively differentiating low and high-grade tumors in 5 min 31 s [94]. Similarly, Sedykh et al. used CS in snapshot CEST for whole-brain APTw imaging in under 2 min with minimal signal loss [78].

Beyond CS and parallel imaging, low-rank reconstruction methods have also been applied to exploit redundancies in the spectral domain of the z-spectrum [95,96,97,98]. These approaches leverage the inherent low-dimensional structure of CEST data to reduce artifacts, improve quantification accuracy, and enable shorter scan times.

These approaches collectively make CEST imaging faster and more practical, though challenges remain, such as trade-offs between speed and artifact levels, potential loss of subtle CEST contrast, and increased computational demands, creating opportunities for emerging technologies like AI to further enhance CEST image reconstruction.

In practice, the acquisition protocol should be designed based on the targeted signal. Thus, frequency offsets, saturation power, and saturation duration should be selected according to the target proton pool. The readout must also balance preservation of CEST contrast with scan time, and care must be taken when using high acceleration factors. Although some reconstruction frameworks can recover images with good structural details, this does not guarantee preservation of CEST contrast. Accelerated strategies using offset reduction, CS, or deep learning are promising; however, newly developed approaches should be tested for stability in phantom and/or volunteer scans before patient imaging in neuroinflammatory studies or other CEST applications.

### 3.5. CEST Post-Processing

The CEST signal is inherently small, typically around 5% of the water signal, and strongly dependent on frequency offset [42]. As such, a series of post-acquisition corrections must be applied to improve data quality and enable accurate quantification. These key post-processing steps include motion correction, $B_{0}$ and $B_{1}$ field inhomogeneity corrections, spectral/spatial denoising, normalization and contrast generation. Therefore, each of these post-processing steps must be handled with care to ensure reliable CEST data for interpretation.

#### 3.5.1. Motion Correction

Motion correction in CEST MRI deals with aligning images from various saturation offsets to reduce artifacts caused by patient movement. Even subtle, sub-voxel head motion during the scan can misalign the stack of saturation-weighted images, leading to distortions in the computed CEST spectra and parametric maps [99]. This issue is particularly critical in neuroinflammatory imaging, where patients (e.g., those with acute stroke or encephalitis) may have difficulty remaining motionless. In such cases, retrospective motion correction becomes essential to avoid artifactual CEST contrast. However, traditional intensity-based MRI registration methods often fail for CEST data because the image intensity systematically changes with saturation frequency and is often very low near the water resonance [99].

To address this, specialized algorithms have been developed. For example, Wech et al. exploited the low-rank structure of the Z-spectral image series to robustly register CEST volumes using the low-rank approximation of the z-spectrum (LRAZ) method [95]. Similarly, Bie et al. proposed a two-step robust principal component analysis (RPCA) to separate and correct bulk and subtle motion in CEST images [100]. Deep Learning-based approaches have also been explored to develop more robust algorithms for motion correction in CEST data [99]. By minimizing motion artifacts, this step ensures that observed CEST signal differences in the brain primarily reflect underlying biochemical processes rather than subject movement.

#### 3.5.2. $B_{0}$ Correction

$B_{0}$ inhomogeneity refers to spatial variation in the main magnetic field that causes the water resonance frequency to shift across the image [101,102]. Even a small frequency offset can misalign the saturation frequency with the intended exchangeable protons, leading to artificial signal asymmetry. In Z-spectrum analysis, this manifests as a shift of the apparent “0 ppm” reference, so that a saturation labeled as 3.5 ppm may not exactly hit the amide proton frequency in certain voxels [101]. This effect is even more pronounced at high magnetic field strengths, and if left uncorrected, $B_{0}$ inhomogeneity can produce spurious CEST contrast [103].

As such, $B_{0}$ mapping and correction are necessary for accurate CEST analysis in neuroinflammation. A common static approach is the Water Saturation Shift Referencing (WASSR) method, which acquires a low-power saturation spectrum around water to directly measure the frequency shift in each voxel [101]. The measured water frequency map is then used to realign each voxel’s Z-spectrum by redefining 0 ppm locally. An alternative is to acquire a field map with a quick gradient-echo scan, but WASSR is tailored to CEST and minimizes interference by applying a mild RF pulse [104]. More recent sequences such as Water Shift And B1 mapping (WASABI) even allow simultaneous $B_{0}$ and $B_{1}$ mapping in about one minute [103]. During CEST acquisitions, $B_{0}$ can drift because of motion and system heating, which leads to residual Z-spectral misalignment. Therefore, dynamic $B_{0}$ correction methods have been shown to outperform static approaches, since static methods assume a temporally stable field that often does not hold during longer scans [105,106].

Generally, after $B_{0}$ correction, the water peak is properly aligned to 0 ppm in each voxel, ensuring that CEST offsets (e.g., amide peak at ~3.5 ppm) are truly on-resonance with their target exchangeable protons. This produces a more specific CEST contrast, which is reflective of underlying changes in pH, protein content, or metabolite concentration in the brain.

#### 3.5.3. $B_{1}$ Correction

$B_{1}$ inhomogeneity refers to the non-uniformity of the transmitted RF field within the imaging tissue, which affects the quality of the measured MRI signal and becomes more pronounced at higher field strengths [107,108]. Variations in $B_{1}$ lead to non-uniform saturation power: some regions receive a stronger or weaker saturation pulse than intended, directly influencing the magnitude of the CEST effect. Since CEST contrast depends on the applied saturation power, spatial $B_{1}$ variations may falsely appear as differences in metabolite concentration, which is not the case [102]. Therefore, $B_{1}$ correction is necessary to eliminate this confounding effect.

One approach is to acquire a $B_{1}$ map using the WASABI sequence and apply it for the $B_{1}$ correction [103]. Papageorgakis et al. proposed an analytical post-processing method that rapidly extracts $B_{0}$ and $B_{1}$ maps from WASABI data, significantly reducing computation time while maintaining accuracy, thus enabling integration of WASABI-based corrections into routine clinical CEST workflows [102]. Zhang et al. also developed a Conditional Variational Autoencoder (CVAE) model for generalized $B_{1}$ correction that generates $B_{1}$-corrected Z-spectra from a single acquisition, enabling accurate CEST quantification across varying $B_{1}$ conditions [108]. Wu et al. proposed a direct saturation-removed omega plot model enabling accurate $B_{1}$ correction from two acquisitions, yielding more homogeneous APTw maps and outperforming conventional interpolation-based methods at 5T [107].

Essentially, $B_{1}$ correction compensates for spatial variations in RF transmit field strength that affect saturation efficiency in CEST MRI. It ensures that observed contrast reflects true molecular differences rather than uneven RF delivery, achieved through approaches such as signal normalization, pulse power adjustment, or incorporating $B_{1}$ variations into signal modeling for accurate quantification.

#### 3.5.4. Denoising

After correcting motion and field inhomogeneities, CEST data still suffer from low SNR. Because the CEST effect is very small relative to the water signal, the Z-spectrum and derived maps are highly sensitive to noise. Spectral and spatial denoising techniques reduce noise in Z-spectra and images while preserving CEST contrast. It involves applying filters across frequency offsets or voxels and/or exploiting data redundancies. A simple approach involves using smoothing filters, such as smoothing spline interpolation, cubic smoothing splines, and Gaussian filters, to reduce oscillations in Z-spectra [97].

However, advanced denoising methods include using low-rank approximations and PCA. In these methods, the set of Z-spectra in a neighborhood or across the whole image is assumed to lie in a low-dimensional subspace, with noise removed by truncating to the major components. Goldenberg et al. applied PCA to arrange noise and signal components in the Z-spectrum curve, achieving robust noise suppression without blurring the true contrast [109]. Other methods use self-similarity, such as a non-local means filter across the image, to average voxels with similar spectral profiles even if they are far apart to enhance SNR. Romdhane et al. developed a hybrid filter combining non-local averaging with anisotropic diffusion specifically to improve pH-weighted CEST in tumors [110]. Chen et al. introduced a machine learning based spatial-spectral redundancy denoising method called “BOOST”, which uses a global low-rank projection combined with local spectral smoothness and non-local spatial similarity constraints for improved accuracy [97].

This denoising step increases effective SNR, which improves the detection of true CEST changes associated with neuroinflammatory diseases.

#### 3.5.5. Normalization Using an Unsaturated Scan

CEST quantification requires normalizing the signal to a reference image acquired without saturation [19]. This unsaturated image represents the full equilibrium water signal and is used to scale the saturation images on a voxel-wise basis. Typically, an additional scan without RF saturation is acquired, either before or after the series of saturation images, and used for normalization [42,52]. This step is essential for correcting coil sensitivity variations, T_1_-weighting, and proton density differences across the brain [101].

Without normalization, regions with inherently lower baseline MRI signals due to $B_{0}$/$B_{1}$ inhomogeneity or receive coil sensitivity could falsely appear to have elevated CEST effects [101]. Normalization by an unsaturated image standardizes the signal, enabling accurate calculation of metrics such as MTRasym. In this way, normalization enhances the reliability of CEST maps by eliminating static intensity biases and enables direct pixel-wise comparisons across the Z-spectrum.

#### 3.5.6. Contrast Generation

The processed CEST data are converted into quantitative contrast maps for interpretation. As discussed earlier several contrast maps can be generated including $MTR_{asym}$, APT#,  NOE#*,* $MTR_{Rex}$, AREX and LD(Δω). These maps allow for distinguishing overlapping signals in neuroinflammation, enabling precise pathology quantification.

Beyond these maps fitting approaches are also used. Lorentzian line-shape fitting can decompose the Z-spectrum into multiple pools (amide, amine, NOE, water direct saturation, etc.), providing pool specific maps [111].

All these steps in the CEST post-processing are very important and should be done diligently in order to obtain a reliable CEST signal for analysis.

### 3.6. Artificial Intelligence (AI) Integration in CEST Imaging

AI shows promise for improving CEST imaging in a variety of aspects [112]. However, the most common AI paradigm, supervised learning, relies heavily on acquired data; therefore, the quality of the data and accuracy of labeling strongly influence prediction uncertainty. This makes data engineering a critical consideration when developing supervised or semi-supervised approaches [113]. In MRI physics, signal sensitivity and image contrast often vary significantly depending on the acquisition parameters, such as echo time, repetition time, saturation power, and offset frequency. Because CEST imaging enables the investigation of different metabolites and their sensitivity to both fast and slow exchange rates, incorporating these priors along with relevant protocol parameters can substantially improve model training.

AI has been increasingly applied in CEST imaging for reconstruction, denoising and advanced postprocessing or quantification tasks, as reviewed by Pan et al. [112]. In the reconstruction domain, Zaiss et al. showed that ultrahigh-field spectral information at 9.4 T can be predicted from 3 T spectra using their deep neural network based CEST (DeepCEST) network, allowing high-field contrast from routine lower-field scans [114]. Subsequently, Glang et al. applied DeepCEST at 3 T and demonstrated that the networks could directly predict Lorentzian fitting parameters from uncorrected spectra with high speed and robustness [115].

Several AI-based studies have exploited redundancy in undersampled CEST datasets to improve reconstruction efficiency and image quality. For instance, Guo et al. demonstrated that correlations across frequency offsets could be harnessed to reconstruct high-quality images from PROPELLER acquisitions [116]. Similarly, Xu et al. introduced a model-based Variational Network (CEST-VN) that accelerates CEST imaging by reconstructing high-quality images from undersampled multi-coil data using 3D spatial-frequential convolutional kernels to capture correlations in the spatial and spectral domains [117]. In addition, Xiao et al. proposed a bidirectional long short-term memory (BiLSTM) framework to infer steady-state spectra from non-steady-state acquisitions, substantially reducing scan duration [118]. They also implemented a sequence-to-sequence (seq2seq) model to generate denser z-spectra from a limited number of acquired images, effectively reducing the number of required frequency offsets and decreasing scan time by up to two-thirds [119].

Other groups have addressed acceleration in CEST imaging through AI-based sampling optimization. Cheema et al. proposed “Fisher offsets” and trained a U-Net to reconstruct full CEST maps from sparsely sampled spectra [84]. Liu et al. combined frequency-offset-dependent sampling with a partially separable network, achieving up to 14-fold acceleration in both healthy and patient cohorts [42]. Yang et al. proposed Attention-Based MultiOffset Deep Learning Reconstruction of Chemical Exchange Saturation Transfer (AMO-CEST), an attention-based network that reconstructs CEST images from undersampled radial acquisitions, preserving APTw and rNOE maps with fewer artifacts [120]. In contrast, Liu et al. developed implicit neural representation combined with explicit sparse prior (INRESP), an unsupervised implicit neural representation that adapts directly to each scan using sparse priors, requiring no training data [121].

Noise and low SNR remain key challenges in CEST imaging. To address this, several groups have proposed deep learning based denoising frameworks. Chen et al. introduced the Denoising CEST Network (DECENT), which learns spatiotemporal correlations to suppress Rician noise across offsets, outperforming conventional filters [122]. Radke et al. trained autoencoders on synthetic Bloch-McConnell phantoms, demonstrating ResUNet based denoisers outperform PCA and block matching combined with 3D filtering (BM3D) across a wide range of noise levels [123]. Kurmi et al. developed a denoising convolutional autoencoder that preserved subtle APTw and NOE contrasts in phantom and tumor datasets [124].

Quantitative modeling remains another area where AI has shown substantial promise in CEST. Kim et al. developed magnetization transfer contrast MR fingerprinting (MTC-MRF) with a deep neural network decoder, enabling disentangling of semisolid MT, APTw, and NOE signals for quantitative mapping [125]. Perlman et al. introduced AutoCEST, a physics-governed differentiable network that co-optimized saturation schedules and reconstruction, reducing acquisition to under 70 s while producing quantitative exchange maps [126]. In oncology application, Perlman et al. further demonstrated deep learning-aided CEST-MRF to quantify tumor pH and protein content after oncolytic virotherapy, providing a sensitive noninvasive biomarker of early apoptosis [127].

Other studies have aimed to improve model fitting itself in CEST analysis. Mohammed Ali et al. introduced single Lorentzian Fitting Network (sLoFNet), which replaced least-squares Lorentzian fitting of WASSR spectra with a neural network, offering faster and more robust $B_{0}$ referencing [128]. Heo et al. presented deep learning extrapolated semisolid magnetization transfer reference (DeepEMR), a method that extrapolates semisolid MT references to isolate clean APTw and rNOE signals with excellent reproducibility [129]. More recently, transformer-based fitting approaches for Bloch-McConnell models have been developed, outperforming traditional gradient solvers highlighting the potential of attention mechanisms for accurate CEST parameter estimation [130].

The clinical utility of AI-driven CEST is evident as DeepCEST predictions were generalized to tumor patients, enabling high-field-like maps without ultrahigh-field scanners [114]. The framework was also validated in both volunteers and tumor patients, showing robustness to noise and $B_{0}$ artifacts [115]. Similarly, DeepEMR improved tumor delineation at higher $B_{1}$ levels, providing superior contrast compared to asymmetry analysis [129].

These applications show how AI has been leveraged to accelerate CEST acquisition, enhance reconstruction, and improve post-processing, advancing the field toward more practical and clinically viable CEST imaging.

### 3.7. Preclinical and Clinical Application of CEST to Neuroinflammation

Neuroinflammation can occur in either acute or chronic forms. In the acute state, it initially serves to protect and repair neural tissue, as seen in cases of stroke. However, conditions that promote chronic neuroinflammation contribute to neurodegenerative diseases, including AD, PD, Huntington’s disease (HD) and ALS [131].

In these conditions, neuroinflammation typically originates within the CNS in response to pathological protein aggregation, neuronal injury, and release of danger-associated molecular patterns (DAMPs) [132]. These endogenous triggers activate microglia, the brain’s resident immune cells, which adopt a pro-inflammatory phenotype, releasing cytokines and reactive oxygen species that cause neuronal damage [133]. Sustained microglial activation leads to a self-reinforcing inflammatory loop that contributes to disease progression.

Although the primary drivers of neuroinflammation in neurodegenerative diseases are intrinsic to the CNS, systemic inflammation can further enhance this process. Peripheral infections or systemic immune activation, such as in HIV-associated neurocognitive disorder (HAND) or sepsis-associated encephalopathy, can lead to infiltration of peripheral immune cells into the CNS. This process begins with microglial activation, releasing pro-inflammatory cytokines that compromise the BBB, allowing peripheral T-cells and macrophages to enter [134,135]. These infiltrating immune cells amplify the inflammatory response [136].

**Figure 4:**
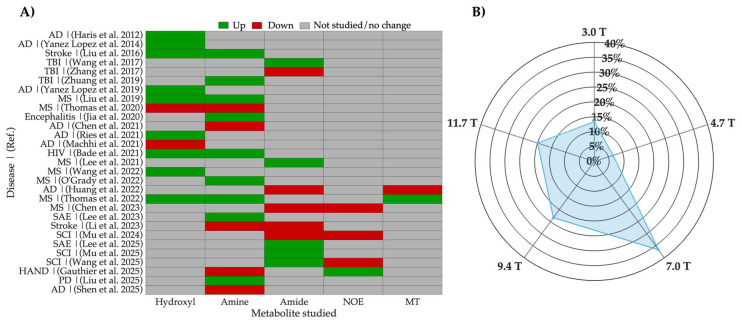
Overview of reviewed CEST MRI studies in neuroinflammatory diseases. (**A**) Distribution of studied metabolite groups: hydroxyl-containing compounds include MI, generic OH, GlucoCEST, 2DG, and DexCEST(OH), while the amine group includes glutamate, GABA, creatine, guanidinium, and antiretroviral drug amines, which was the most frequently reported [23,29,32,57,137,138,139,140,141,142,143,144,145,146,147,148,149,150,151,152,153,154,155,156,157,158,159,160,161]. (**B**) Percentage distribution of field strengths across studies, showing that 80% were performed at high fields (7 T or more), demonstrating the strong preference for high -field application [23,29,32,137,138,139,141,142,143,144,145,146,147,148,150,152,153,154,155,156,157,158,159,160].

In this section, we review diseases where CEST imaging has been used to detect neuroinflammation, with findings validated through histological studies and immunochemistry assays identifying pro-inflammatory cytokines such as interleukin-1 beta (IL-1β) and TNF-α, or activated immune cells including ionized calcium-binding adapter molecule 1-positive (Iba-1^+^) microglia and cluster of differentiation 68-positive (CD68^+^) macrophages. Figure 4 presents an overview of the reviewed CEST MRI studies in neuroinflammatory diseases while Table 1 summarizes the preclinical and clinical investigations, including the examined CEST parameters, validation biomarkers, and saturation conditions.

#### 3.7.1. Primary Neuroinflammatory Diseases

##### Multiple Sclerosis and Encephalitis

CEST imaging has been extensively used in preclinical and clinical studies of MS to map inflammatory metabolic changes. In a study by Liu et al., CEST MRI was used to detect subtle pathological alterations in the cerebellum and brainstem of Experimental Autoimmune Encephalomyelitis (EAE) mice at a very early stage (6 days post-induction), before conventional MRI showed any abnormalities [144]. They targeted hydroxyl protons (e.g., from glycans and lactate) at 1 ppm and amine protons at 2 ppm. Histogram-based analysis of the Z-spectra revealed heterogeneous signal distributions and reduced peak intensities in EAE mice compared to naive controls, with significant increases at both frequency offsets. These changes were validated by Iba-1 immunofluorescence, confirming microglial activation. Thomas et al. also investigated cervical lymph nodes in EAE mice using CEST imaging at multiple frequency offsets (1.6, 3.2, and 5.2 ppm) [32]. They found elevated CEST signal intensity that correlated with immune activation markers detected via flow cytometry, including upregulated expression of CD11b, CD86, and IL-17 in both lymph nodes and central nervous system tissue. Additionally, matrix-assisted laser desorption/ionization (MALDI) imaging revealed increased levels of alanine, lactate, and other metabolites in the lymph nodes of EAE mice.

Wang et al. employed Dextran-enhanced CEST (DexCEST) at +1.0 ppm (OH protons from dextran) in EAE mice, observing upregulated signals, which were validated by Gd-enhanced MRI and fluorescence microscopy (Dex3-FITC/Dex40-TRITC) assessing BBB permeability [145]. In the only clinical study identified where CEST imaging was applied to assess neuroinflammation in MS, O’Grady et al. quantified glutamate at 3.0 ppm in relapsing-remitting MS patients and healthy controls using AREX. After removing MT, spillover, and relaxation effects that are altered in MS due to myelin loss and increased water content, AREX values in WM lesions remained higher than in normal-appearing WM. These corrected values were consistently shifted toward positive contrast, indicating that the residual exchange-dependent signal reflects a biochemical alteration rather than a structural effect. The elevation was consistent with glutamate dysregulation known to occur during neuroinflammatory activity, supported by prior MRS and histopathology evidence. This demonstrates the potential of GluCEST to provide complementary information relevant to neuroinflammatory pathology [146]. Hence, CEST should be considered when metabolic characterization is needed to complement structural MRI, particularly in scenarios where inflammatory or metabolic alterations are suspected but conventional imaging lacks specificity.

Thomas et al. implemented on-resonance variable delay multiple pulse (onVDMP) CEST imaging to target fast-exchanging protons (e.g., glutamate, creatine, lactate) in EAE mice. They observed decreased signals in the corpus callosum, hypothalamus, and third ventricle as early as 12–13 days post-induction, preceding paralysis onset. These early alterations were not detected with CW or APTw CEST. Histological validation confirmed astrogliosis (GFAP) and microglial activation (CD11b/CD68) without demyelination or axonal loss [147].

APTw imaging has been employed to monitor demyelination and remyelination in the corpus callosum of cuprizone-induced rat models. The amide signal increased during demyelination and returned toward control levels with remyelination [148]. Chen et al. demonstrated decreased rNOE during demyelination but reversed with remyelination; however, the amide signals remained low even after remyelination [149].

In encephalitis, Jia et al. combined preclinical and clinical studies targeting glutamate at 3.0 ppm. They observed elevated GluCEST signals in encephalitis that increased with disease progression and decreased after intravenous immunoglobulin treatment. The consistency between clinical and preclinical findings supported the interpretation that the observed treatment effects reflected a reduction in neuroinflammation and glutamate accumulation [29].

#### 3.7.2. Inflammatory-Related Disorders

##### Alzheimer Disease

In AD, all reported CEST imaging studies for neuroinflammation to date have been conducted exclusively in preclinical models. Yanez Lopez et al. examined hydroxyl proton CEST signals in the range of 0.4−0.8 ppm following unilateral intra-hippocampal injection of lipopolysaccharide (LPS) in amyloid precursor protein/presenilin 1 (APP/PS1) transgenic and wild-type mice. Increased signals were observed on the LPS-injected side and with neuroinflammation inferred due to prior ^1^H-MRS data showing elevated MI [139]. The same group subsequently investigated MI CEST following intracerebral LPS injection in APP/PS1 transgenic and wild-type mice and again observed increased CEST signals in the group that showed strong IBA-1 microglial activation, suggesting that the CEST contrast was sensitive to the inflammatory response [137]. Similarly, Haris et al. examined MI hydroxyl protons at 0.6 ppm in APP-PS1 mice versus wild-type controls and found increased CEST signals at the target offset. This was confirmed by a 51% rise in MI/Cr from ^1^H-MRS and enhanced GFAP expression indicating astrocyte proliferation [23].

Shen et al. applied glutamate- and gamma-aminobutyric acid (GABA)-weighted CEST imaging at 3.0 ppm and 2.75 ppm, respectively, together with ^1^H-MRS to monitor riluzole treatment in 3xTg AD mice [138]. Untreated mice showed reduced Glu/GABA levels, while riluzole-treated animals exhibited increased signals that correlated with reduced amyloid-beta (Aβ), tau, GFAP immunoreactivity and enhanced neuronal survival. These findings indicate the restoration of Glu/GABA homeostasis alongside attenuation of neuroinflammatory activity. Huang et al. developed a deep neural network based DeepCEST to assess amide signals and MT effects in 5xFAD AD mice and age-matched controls. They observed decreased amide signals in both central and anterior brain slices, with additional MT signal reduction that were more pronounced in the anterior slice. 6E10 immunohistochemistry confirmed elevated amyloid-β plaque deposition [140]. In the work of Chen et al., creatine at 2 ppm in APP and Tau AD models was examined, where decreased CEST signals were attributed to intracellular pH changes associated with neuroinflammation. This interpretation was supported by elevated GFAP and IBA-1 and by the presence of weak APP plaques and tau tangles, although no changes in Cr/PCr were detected by ^1^H-MRS and P-MRS [141].

In addition, Ries et al. used GlucoCEST imaging to evaluate BBB integrity in young 5xFAD mice following treatment with human recombinant Annexin A1 (hrANXA1), a pro-resolving anti-inflammatory mediator [142]. Untreated 5xFAD mice showed significantly higher GlucoCEST signals than both wild-type and hrANXA1-treated 5xFAD mice at baseline and all time points. hrANXA1-treated mice also showed decreased pro-inflammatory cytokines, including IFNγ, TNFα, and IL-1β. Machhi et al. also employed GlucoCEST imaging to assess hippocampal glucose uptake in APP/PS1 transgenic mice, including cohorts receiving adoptive transfer of Aβ-specific Th1 or Th17 effector T cells [143]. The CEST-derived contrast between 0.8 and 2.2 ppm was significantly reduced in APP/PS1 mice compared to wild-type controls, with further reductions of 87% in the Aβ-Th1 group and 67% in the Aβ-Th17 group. These changes were interpreted as impaired glucose uptake linked to heightened neuroinflammation. This interpretation was supported by immune profiling, which revealed elevated IFNγ, TNFα, and IL-17 levels, and reactive Iba1^+^ microglial morphology.

##### Traumatic Brain Injury (TBI)

Preclinical CEST applications in TBI models have demonstrated sensitivity to acute and subacute inflammatory changes. Zhuang et al. applied GluCEST in a TBI rat model and observed increased signal intensity in the injured cortex and ipsilateral hippocampus, which corresponded with elevated IL-6 and TNF-α cytokine levels in the same regions [150]. Wang et al. investigated APTw in TBI rats and found increased APTw signals in perilesional areas at days 3–7 post-TBI, attributed to glial activation [57]. Treatment with pinocembrin reduced these signals, indicating diminished inflammation. Validation included Iba-1 and GFAP immunofluorescence for microglia and astrocytes, as well as cresyl violet staining for neuronal survival. In addition, Zhang et al. applied APTw imaging in TBI rats and observed decreased signals in the ipsilateral cortex and hippocampus at 1–6 h post-injury due to acidosis [151]. This was followed by marked peri-lesional increases at 2–3 days, indicative of inflammation, confirmed by Iba-1+ microglial and GFAP+ astrocytic activation.

##### Stroke

Preclinical CEST studies in ischemic stroke models emphasize therapeutic monitoring and contrast agents. Liu et al. evaluated hydroxyl (+1.0 ppm) and amine (+2.0 ppm) CEST signals from citicoline-loaded liposomes in a rat stroke model, and they observed post-injection signal increases in ischemic regions [154]. These findings, confirmed by T_2_w hyperintensity, fluorescence microscopy, and VCAM-1 expression with binding specificity, revealed citicoline as a label-free CEST contrast agent. Li et al. explored amide protons and guanidinium amine protons in cerebral ischemia–reperfusion injury (CIRI) rats and found that the CEST signal decreased as early as 2 h post-CIRI, consistent with pH changes after ischemia. Ischemic stroke induces significant pH reductions in affected brain tissue due to metabolic acidosis, detectable by CEST MRI, which sensitively maps pH changes in preclinical models. These alterations reflect tissue damage and neuroinflammation, aiding diagnosis and therapeutic monitoring [162,163,164,165,166]. Following melatonin treatment, both Amide-CEST and guanidinium CEST (GuanCEST) signals increased in the ischemic cortex and striatum, with immunofluorescence and Western blotting showing increased M2 microglial markers (Arg1, CD206) and decreased IL-1β expression compared with untreated CIRI rats [155]. In addition, clinical APTw imaging has been demonstrated to be sensitive to physiological changes associated with ischemic stroke, as shown by Heo et al., where APTw showed potential for assessing stroke severity, treatment response, and outcome prediction [167].

##### Spinal Cord Injury (SCI)

CEST applications in SCI models detect inflammation and treatment effects. Mu et al. examined targeted amide protons and NOE signals at −1.6 ppm in a rat model of traumatic SCI and it turned out there were marked changes within the first week post-injury. Specifically, NOE decreased while amide signals increased in segments rostral to the injury epicenter. These changes showed strong spatial correspondence with increased PET-TSPO uptake and were validated by post-mortem Iba-1 and GFAP immunostaining, which confirmed elevated microglial and astrocytic activation [157]. Mu et al. further studied amide at 3.5 ppm in SCI rats and found it increased in the injured cord at week 1 but decreased with riluzole treatment. This was validated by lowered Iba-1, unchanged GFAP, increased LFB staining, and improved BBB scores, indicating reduced neuroinflammation [156]. Wang et al. also investigated amide and NOE at −1.6 ppm in injured spinal dorsal nerve roots in squirrel monkeys. They observed amide upregulation and NOE downregulation specific to the injured areas, findings attributed to inflammation, demyelination, and fiber degeneration [158].

##### Sepsis-Associated Encephalopathy (SAE)

CEST has shown promise in detecting neuroinflammation in infectious and systemic inflammatory models. In SAE, Lee et al. examined glutamate in LPS-induced SAE rats, finding dose-dependent GluCEST increases, which correlated with elevated hippocampal glutamate on ^1^H-MRS and Iba-1/NeuN/DAPI staining indicating neuroinflammation [152]. Recent work by this group focused on APTw in LPS-induced SAE rats. They observed hippocampal APTw upregulation, which was used to characterize neuroinflammation, as previous studies had shown microglial activation [153].

##### Other Neuroinflammation Applications

There has also been less common application of CEST to study neuroinflammation conditions like HAND and PD. Bade et al. used hydroxyl (~1 ppm) and amino (~2 ppm) CEST signals to monitor lamivudine and emtricitabine biodistribution for HIV treatment. After inducing neuroinflammation with LPS in mice, they showed that amine and hydroxyl signals correlated with plasma and brain drug concentrations, thereby validating CEST imaging as a potential marker of antiretroviral (ARV) delivery [160]. Gauthier et al. conducted a longitudinal preclinical study in HIV-1-infected CD34-NSG humanized mice to assess neurometabolic alterations using CEST MRI at 2 ppm (creatine), 3 ppm (glutamate), and −3.5 ppm (NOE) [159]. At 6 weeks post-infection, creatine levels were significantly reduced in the cortex and hippocampus, while NOE signals increased in the cortex. By 12 weeks, vehicle-treated mice exhibited further decreases in glutamate across the cortex, hippocampus, and piriform cortex, with ART partially restoring these glutamate deficits in the cortex and hippocampus. Similarly, creatine reductions extended to the piriform cortex and thalamus at 12 weeks, with ART promoting recovery in most regions except the cortex. In contrast, the elevated NOE signals in the cortex and thalamus were not reversed by ART. These imaging findings were confirmed by histological assessments showing reduced HIV-1 p24+ cells and attenuated glial activation (Iba-1, GFAP, HLA-DR) in ART-treated animals, supporting the association of CEST-detected metabolic changes with neuroinflammation and viral activity.

In the case of PD, Liu and his group recently carried out a clinical study comparing PD patients and healthy controls, focusing on glutamate at 3.0 ppm [161]. They observed elevated striatal and thalamic glutamate levels in PD, and these increases correlated with the United Parkinson’s Disease Rating Scale (UPDRS) III motor scores. Structural MRI showed reduced left pallidum volume. The glutamate elevations were interpreted as consistent with abnormal glutamatergic activity linked to neuroinflammation and dopaminergic dysfunction. 

Although direct studies confirming CEST MRI for neuroinflammation in brain tumors and HD are scarce, their pathophysiology supports its potential. Brain tumors like gliomas feature uncontrolled proliferation, BBB disruption, and inflammation driven by tumor-associated macrophages and pro-inflammatory mediators [168]. CEST can track associated protein changes, such as amide proton shifts for aggregates or mobile proteins, and metabolite alterations like glutamate for excitotoxicity in these diseases [169]. Application of CEST to study brain tumors is the most popular among the various applications of CEST.

Zhou et al. showed that endogenous APTw contrast could visualize elevated mobile protein content and produce a well-defined hyperintense tumor region compared with conventional T_1_, T_2_, and diffusion-weighted imaging [73]. Warnert et al. applied pulsed APTw CEST with full-tumor coverage in non-enhancing gliomas, revealing that Lorentzian-difference metrics could distinguish tumor from normal-appearing WM [170]. Xu et al. also compared APTw with diffusion kurtosis imaging in 51 glioma patients and found that APTw contrast provided superior discrimination of tumor grade and IDH mutation status relative to DKI-derived metrics, supporting APTw as a sensitive marker of tumor molecular composition and malignancy [171].

HD involves CAG expansions in the HTT gene, leading to mutant huntingtin aggregates, neuronal death in the striatum, and chronic neuroinflammation via microglial activation and cytokine release [172,173]. CEST MRI has been applied to investigate metabolic and protein alterations in HD, particularly using gluCEST) to detect metabolic changes associated with disease progression [174]. Longitudinal multimodal MRI integrating gluCEST, diffusion tensor imaging, and magnetization transfer imaging has further revealed early microstructural and metabolic abnormalities in white and gray matter regions especially within the corpus callosum, striatum, and frontal cortex preceding neurodegeneration [175]. These findings show the potential of CEST MRI to sensitive metabolite changes linked to neuroinflammatory mechanisms in HD.

### 3.8. Potential of CEST and AI in Investigating Neuroinflammation

Given the sensitivity of CEST to chemical signatures associated with neuroinflammation, AI can play a crucial role in advancing diagnostic accuracy and precision medicine by enabling molecular subtyping and monitoring treatment response in neuroinflammatory conditions.

Several transferable AI methodologies have already been developed in the CEST domain, providing frameworks that can be adapted to neuroinflammatory contexts. In disease characterization, Sartoretti et al. demonstrated the application of CEST imaging for differentiating gliomas [176], while Wu et al. incorporated AI techniques to classify diffuse gliomas, though accuracy was limited in distinguishing oligodendrogliomas from astrocytoma [177]. Bie et al. also applied convolutional neural networks (CNNs) to preclinical breast cancer CEST data [178], and Wei et al. employed radiomics to predict metastasis in rectal cancer [179]. These studies highlight methodological innovations that can be adapted to neuroinflammatory contexts.

With respect to molecular subtyping, several groups have demonstrated the value of AI-assisted CEST imaging in defining molecular heterogeneity. For instance, Zhuo et al. demonstrated that radiomics features derived from APTw imaging, combined with machine learning classifiers, could predict H3K27M mutations in brainstem gliomas [180]. Hagiwara et al. used unsupervised clustering and support vector machine (SVM) algorithms applied to multiparametric CEST data, including amine and APTw signals, to non-invasively stratify diffuse gliomas by isocitrate dehydrogenase (IDH) mutation status, thereby capturing metabolic heterogeneity [181]. Yuan et al. trained a CNN on APTw-CEST data to classify IDH-mutant versus wild-type gliomas, extracting subtle spectral differences between molecular subtypes [182]. Chu et al. introduced a Dual-Aware deep learning framework that uses APTw images for robust IDH genotyping in gliomas [183].

In the area of treatment response monitoring, Paprottka et al. conducted objective evaluations using quantitative CEST metrics, and Guo et al. applied a ResNet-based deep learning model to track therapeutic response patterns [184,185]. In addition, free water imaging as a complementary biomarker can be combined with CEST and integrated into AI-driven pipelines for improved assessment of therapeutic effects in neuroinflammatory diseases [186,187].

### 3.9. High Field CEST MRI for Neuroinflammation Assessment

Out of the reviewed papers, we identified 24 studies that employed high field strengths (≥7 T, up to 11.7 T) for CEST imaging [23,29,32,137,138,139,141,142,143,144,145,146,147,148,150,152,153,154,155,156,157,158,159,160]. These field strengths were used for these studies because they enhance CEST contrast through increased chemical shift dispersion, higher SNR, and improved sensitivity to subtle metabolic and pH-related alterations [188]. Such advantages make high-field CEST imaging particularly well suited for detecting neuroinflammatory changes in molecular pools associated with glutamate, creatine, amide, and hydroxyl protons.

However, achieving reliable and reproducible outcomes at high field requires correction of $B_{0}$ and $B_{1}$ inhomogeneities, mitigation of motion artifacts, and confounding factors such as MT and NOE, which become more pronounced with increasing field strength. Most studies reviewed incorporated $B_{0}$ and $B_{1}$ inhomogeneity correction methods to mitigate these effects. Future efforts should prioritize harmonizing high-field CEST protocols with clinical 3 T systems to ensure that biomarkers identified at 7–11.7 T can be validated, standardized, and ultimately translated into clinical practice for the assessment of neuroinflammation.

## 4. Discussion

In this review, we presented applications of CEST imaging, primarily preclinical with a few clinical studies, as a non-invasive technique for detecting neuroinflammation across a range of neurological conditions, including MS, AD, TBI, stroke, SCI. Across these studies, CEST has been consistently applied to characterize metabolic changes associated with inflammatory processes, with histological validation through markers such as Iba-1^+^ microglia, GFAP^+^ astrocytes, and pro-inflammatory cytokines (IL-1β, TNF-α).

Among the targeted metabolites, glutamate (~3.0 ppm via GluCEST) has emerged as the most frequently studied, reported in MS, AD, TBI, SAE, encephalitis, HIV, and PD. Amide protons (~3.5 ppm via APTw) have also been widely investigated, particularly in MS, TBI, stroke, SCI, and SAE, where they serve as sensitive indicators of pH alterations and protein content changes. Hydroxyl protons (0.6–1.0 ppm) from metabolites such as MI, glycans, and lactate have been prominent in MS, AD, and stroke models, often linked to glial activation and altered glycolytic metabolism. Additional exploited contrasts included amine protons (~2.0 ppm) in MS and stroke, creatine (~2.0 ppm) in AD, HIV, and MS, and NOE signals (−1.6 to −3.5 ppm) in SCI and HAND. This broad metabolic coverage demonstrates CEST’s versatility in probing multiple biochemical pathways implicated in neuroinflammation.

Our observations indicate that CEST has been extensively utilized as a biomarker for neuroinflammation in numerous neurodegenerative disease models, with the majority of studies conducted in preclinical settings using rodent or primate models. Clinical translation remains limited, with reported applications primarily in MS, encephalitis, and PD. Nevertheless, in humans, many neurodegenerative diseases, including AD, PD, ALS, and HD originate with primary CNS-mediated inflammation driven by protein aggregation and danger-associated molecular patterns (DAMPs). This acute microglial activation can progress into chronic, self-perpetuating inflammation, while in conditions such as HAND and SAE, systemic immune activation and peripheral immune cell infiltration further exacerbate CNS injury. CEST’s ability to detect pH changes, metabolite shifts, and microglial or astrocytic activation makes it particularly suited for monitoring both early and sustained inflammatory activity in humans. While Saiyisan et al. reviewed CEST applications in neurodegenerative diseases, the metabolic alterations they highlighted provide a rationale for extending CEST applications toward investigating neuroinflammatory mechanisms in these disorders [48].

Several technical and biological advantages position CEST favorably among molecular imaging techniques. Unlike PET or SPECT, it avoids ionizing radiation, allowing for safe, repeated longitudinal assessments. Compared to MRS, CEST offers higher spatial resolution and the capacity to simultaneously quantify multiple exchangeable proton pools with greater spectral specificity. Furthermore, most CEST studies exploit endogenous metabolites, eliminating the need for exogenous contrast agents [14]. In cases where exogenous compounds are employed, such as glucose in GlucoCEST for assessing BBB integrity or altered glucose uptake, the agents are biocompatible and metabolically relevant, in contrast to gadolinium-based MRI agents, which carry risks of nephrotoxicity and tissue deposition [189].

Collectively, these features make CEST a powerful and versatile imaging strategy with strong potential for integration into clinical workflows, enabling early diagnosis, disease monitoring, and personalized therapeutic assessment in neuroinflammatory disorders.

### 4.1. Limitations and Translational Challenges

Despite its promise, the application of CEST imaging for neuroinflammation faces several technical and methodological challenges that limit its widespread adoption. CEST contrast is highly sensitive to $B_{0}$ and $B_{1}$ inhomogeneities, motion, temperature, and acquisition parameters, complicating reproducibility across scanners and imaging sites even at 3 T.

A major limitation arises from the spectral proximity of many commonly targeted frequency offsets, such as glutamate (~3.0 ppm), amine, and glucose hydroxyl protons, to the water resonance. This makes spectral specificity highly dependent on magnetic field strength. At lower magnetic fields (≤3 T), achieving sufficient separation from the water peak is difficult, favoring the use of ultra-high-field systems (7 T, 9.4 T). However, higher fields introduce their own challenges, particularly pronounced $B_{0}$ and $B_{1}$ inhomogeneities, necessitating robust correction strategies to ensure accurate quantification.

Another key limitation is the lack of standardized acquisition protocols. Parameters such as $B_{1}$ amplitudes, saturation durations, and pulse schemes vary considerably across studies, complicating comparisons. While consensus guidelines exist for APTw imaging in tumors [190], similar standardization has not yet been established for neuroinflammation. In addition, there is no universally accepted framework for CEST data analysis, further limiting reproducibility.

Methodological challenges also include the absence of fast CEST acquisition strategies, such as optimized k-space sampling combined with advanced reconstruction methods, which could substantially reduce scan times and improve clinical feasibility. Additional confounders include motion sensitivity, susceptibility artifacts in certain brain regions, and partial volume effects, all of which can confound signal interpretation. Addressing these limitations is essential for improving reproducibility, sensitivity, and translational potential of CEST for neuroinflammation imaging.

### 4.2. Future Directions and Recommendations

Future research should prioritize the development of standardized acquisition protocols and multisite data harmonization for metabolites relevant to neuroinflammation. Harmonized acquisition parameters—such as saturation power, duration and frequency offsets —should be implemented to ensure reproducibility across scanners and magnetic field strengths. The development of standardized phantoms and cross-calibration frameworks will facilitate quantitative comparison of CEST metrics between sites. Moreover, the adoption of open-source preprocessing pipelines, AI-driven normalization tools, and shared reference datasets can mitigate inter-site variability and enhance model generalizability. Establishing consensus guidelines, similar to existing APTw tumor imaging standards [190] and along with benchmark datasets developed through collaborative consortia will accelerate validation efforts and promote the clinical translation of CEST MRI to serve as a reliable biomarker for neuroinflammation in both neurological and psychiatric disorders.

Advancing spectral specificity at clinical field strengths (3 T and below) is another critical objective. Potential approaches include advanced water suppression strategies, optimized saturation schemes, or multi-pool Lorentzian fitting to improve separation of metabolite signals from direct water saturation. In parallel, robust $B_{0}$ and $B_{1}$ inhomogeneity correction methods, such as parallel transmit shimming or $B_{0}$ field mapping-based post-processing, should be routinely incorporated, particularly at ultra-high field.

The integration of fast CEST acquisition methods that incorporate strategic sampling techniques paired with CS or deep learning reconstruction, could be explored to substantially reduce scan times without compromising sensitivity. Such undersampling approaches could be guided by prior knowledge of CEST data, such as Lorentzian-optimized frequency offset–dependent (FOD) sampling, which prioritizes offsets with the highest contrast-to-noise ratio for the metabolites of interest. In parallel, reconstruction algorithms should be designed to exploit the low-rank nature of multi-offset CEST spectral-spatial data, incorporating constraints such as spectral smoothness, sparsity, and temporal redundancy to enhance noise robustness and preserve subtle contrast changes. Combining these optimized acquisition strategies with model-based or learning-based reconstruction frameworks could enable high acceleration factors while maintaining quantitative accuracy, ultimately making CEST more practical for routine neuroinflammation imaging in clinical settings.

Finally, longitudinal and treatment-monitoring studies should be expanded to assess CEST’s responsiveness to therapeutic interventions in neuroinflammatory disorders. Hybrid imaging approaches, such as combining CEST with PET, diffusion MRI, or quantitative susceptibility mapping, may also provide complementary metabolic and microstructural information, further improving diagnostic accuracy. Together, these advances will be crucial for translating CEST from predominantly preclinical research to widespread clinical adoption in neuroinflammation imaging.

## 5. Conclusions

CEST imaging is a powerful molecular MRI technique for probing neuroinflammatory processes with high biochemical specificity. By sensitively detecting alterations in pH, metabolites, and protein content, CEST offers unique insights into neuroinflammation beyond conventional imaging. Preclinical studies have demonstrated its ability to detect inflammatory responses in various neurological disorders, corroborated by histology, immunohistochemistry, and spectroscopy. However, clinical translation remains limited by technical challenges such as sensitivity to field strength, motion artifacts, quantification variability, and acquisition time. Standardization of acquisition protocols, multisite harmonization, and robust $B_{0}$/$B_{1}$ correction strategies are essential for reproducibility. The integration of AI for denoising, spectral prediction, and quantitative mapping further enhances clinical applicability. Future efforts should focus on validating CEST biomarkers through histopathological and multimodal correlations, accelerating translation from preclinical to clinical settings. With coordinated standardization and validation, CEST imaging has the potential to become a reliable, noninvasive biomarker for monitoring neuroinflammation, bridging preclinical insights to clinical application and enabling new approaches to track disease progression and therapeutic response.

## Figures and Tables

**Table 1:** Summary of preclinical and clinical CEST MRI studies in Neuroinflammation, showing investigated CEST parameters, validation biomarkers, and saturation conditions.

Diseases	CEST Parameters	Blood/Tissue Biomarkers	Saturation Parameters	Clinical/Preclinical	Reference
AD	Hydroxyl protons at 0.6 ppm (↑)	Iba1+ (strong response)	$B_{0}$ $=\;9.4\;T;\;B_{1}$ = 0.9 μT, T_sat_ = 1.6 s Offsets: ±4 ppm (0.2 ppm steps)	Preclinical	[137]
AD	Glu (3.0 ppm) ↓, (2.75 ppm) ↓; Glu and GABA levels (↑) after riluzole	1H-MRS: Glu, GABA (↑) after riluzole; IHC: Aβ, tau, GFAP (↓) after riluzole Nissl: neuronal survival (↑)	$B_{0}$ $=\;7\;T;\;B_{1}$ = 4 μT; 2 s CW; GRE readout; Offsets: ±5 ppm (0.1 ppm steps)	Preclinical	[138]
AD	Hydroxyl protons 0.4–0.8 ppm (↑)	Inferred neuroinflammation due to elevated MI in prior ^1^H-MRS studies	$B_{0}$ $=\;9.4\;T;\;B_{1}$ = 0.9 μT; T_sat_ = 1.6 s; Offsets: ±4 ppm	Preclinical	[139]
AD	APTw at 3.5 ppm (↓); MT contrast (↓); rNOE unchanged;	6E10 IHC: Aβ plaque (↑)	$B_{0}$ $=\;3\;T;\;B_{1}$ = 0.6 μT; CW; T_sat_ = 3 s; Offsets: ±20 ppm	Preclinical	[140]
AD	Cr at 2 ppm (↓)	^1^H-MRS and ^31^P-MRS: Cr/PCr (no change); GFAP and IBA1 activation (stronger in APP vs. Tau)	$B_{0}$ $=\;11.7\;T;\;B_{1}$ = 2 μT; T_sat_ = 1 s	Preclinical	[141]
AD	MI at 0.6 ppm (↑)	^1^H-MRS: MI/Cr ↑ 51%; GFAP IHC: astrocyte proliferation	$B_{0}$ $=\;9.4\;T;\;B_{1}$ = 75 Hz; T_sat_ = 5 s; Offsets: 0–2 ppm (step = 0.1 ppm)	Preclinical	[23]
AD	GlucoCEST (0.8–2.2 ppm): ↑ before treatment, ↓ after hrANXA1 treatment,	BBB via Evans blue; hrANXA1: ↓ TNFα, IFNγ; ↑ IL-10; ↓ CD3+ T-cell, Aβ40, and p-tau, and ↑ IDE, neprilysin	$B_{0}$ $=\;9.4\;T;\;B_{1}$ = 1.6 µT; T_sat_ = 3 s; Offsets: ±3.2 ppm (17 offsets)	Preclinical	[142]
AD	2DG-CEST (0.8–2.2 ppm); ↓ %ΔMTR in APP/PS1; ↓ Aβ-Th1 (87%) and Aβ-Th17 (67%) groups	↑ IFNγ, TNFα, and IL-17; Iba1^+^ microglia activation; ↑ iNOS, ↓ Arg1 (M1 shift); ↓ Foxp3, Il10, Il13 (anti-inflammatory genes)	$B_{0}$ $=\;7\;T;\;B_{1}$ = 3 µT; T_sat_ = 1 s Offsets: −1600 to 1600 Hz in steps of 80 Hz.	Preclinical	[143]
MS	Hydroxyls (1 ppm ↑); Amines (2 ppm ↑)	IBA1 immunofluorescence (↑); Gd-enhanced T_1_w MRI confirmation	$B_{0}$ $=\;11.7\;T;\;B_{1}$ = 1 μT; T_sat_ = 2.5 s; Offsets: ±4 ppm; (0.25 ppm steps)	Preclinical	[144]
MS	CEST signals at 1.6, 3.2, and 5.2 ppm (↑)	Flow cytometry: CD11b, CD86, IL-17a (↑); MALDI: ↑ alanine, lactate, malate, phenylalanine	$B_{0}$ $=\;11.7\;T;\;B_{1}$ = 1 μT; T_sat_ = 3 s Offsets: 0.4–6.0 ppm (0.4 ppm steps)	Preclinical	[32]
MS	DexCEST at +1.0 ppm (OH ↑)	Gd-MRI (structural); Fluorescence (Dex3-FITC, Dex40-TRITC): BBB permeability	$B_{0}$ $=\;11.7\;T;\;B_{1}$ = 1.8 μT; T_sat_ = 3 s; Offsets: ±3 ppm; (31 offsets, 0.2 ppm steps)	Preclinical	[145]
MS	Glu at 3.0 ppm (↑)	↑ Glutamate in WM lesions: correlated with EDSS and cognitive decline	$B_{0}$ = 7 T; *B*rms = 1.97 μT; T_sat_ = 670 ms pulse train; Offsets: ±5 ppm (43 offsets)	Clinical	[146]
MS	onVDMP: ↓ signal in corpus callosum, hypothalamus, and 3rd ventricle (early EAE). APTw: No significant change	GFAP (↑), CD11B/CD68 (↑); astrogliosis without demyelination	$\mathrm{onVDMP}:\;B_{0}$ = 11.7 T, $B_{1}$ = 46.8 μT, 32 pulses; APTw CEST: $B_{1}$ = 1 μT, T_sat_ = 3 s; Offsets: ±8 ppm (0.4 ppm steps)	Preclinical	[147]
MS	APTw at 3.5 ppm ↑ in DEM; ↓ in REM	TEM and Black-Gold II staining: showing DEM and REM	$B_{0}$ $=\;7\;T;\;B_{1}$ = 2.3 mT T_sat_ = 5 s; Offsets: ±6 ppm (0.5 ppm steps)	Preclinical	[148]
MS	rNOE at −3.5 ppm (↓ in DEM, recovered in REM); Amide at +3.5 ppm (↓ in DEM, remained low in REM)	FluoroMyelin and MBP: ↑ week 8, and ↓ week 14	$B_{0}$ $=\;3\;T;\;B_{1}$ = 0.8 μT; T_sat_ = 3 s; Offsets: ±13 ppm;	Preclinical	[149]
TBI	Glu at 3.0ppm (↑)	IL-6 and TNF-α (↑)	$B_{0}$ $=\;7\;T;\;B_{1}\;$= 5.9 μT; T_sat_ = 2 s; Offsets: ±5 ppm	Preclinical	[150]
TBI	Amide at 3.5 ppm (↑ day 3–7); ↓ with pinocembrin—↓ inflammation	Iba1 and GFAP IHC; Cresyl violet (neuron survival)	$B_{0}$ $=\;4.7\;T;\;B_{1}$ = 1.3 μT; T_sat_ = 4 s; Offsets: ±3.5 ppm	Preclinical	[57]
TBI	Amide at 3.5 ppm (↓ 1–6 h; ↑ 2–3 days peri-lesional)	Iba1+, GFAP+ glial activation (↑ 3 days)	$B_{0}$ $=\;4.7\;T;\;B_{1}$ = 1.3 μT; T_sat_ = 4 s; Offset: ±3.5 ppm	Preclinical	[151]
Encephalitis	Glu at 3.0ppm (↑; ↓ after treatment)	Preclinical: S. aureus–induced microgliosis & astrogliosis; Clinical: ↑ CSF white blood cells	$\mathrm{Preclinical}:\;B_{0}$ = 7 T; $B_{1}$ = 3.6 µT, T_sat_ = 2 s;$\mathrm{Clinical}:\;B_{0}$ = 3 T; $\;B_{1}$ = 5.9 µT; T_sat_ = 2 s Offsets for both: ±5 ppm (0.2 ppm steps)	Both	[29]
SAE	Glu at 3.0ppm (↑)	^1^H-MRS: Glu (↑); Iba-1, NeuN, DAPI	$B_{0}$ $=\;7\;T;\;B_{1}$ = 3.6 μT; Tsat = 1 s; Offsets: ±6 ppm (0.5 ppm steps)	Preclinical	[152]
SAE	Amide at 3.5 ppm (↑) hippocampus)	Neuroinflammation in LPS-induced SAE	$B_{0}$ $=\;7\;T;\;B_{1}$ = 2.3 μT; T_sat_ = 5 s; Offsets: ±6 ppm (0.5 ppm steps)	Preclinical	[153]
Ischemic Stroke	OH at +1.0 ppm and NH_2_ at +2.0 ppm from CDPC (↑ after injection)	↑ Fluorescence and T_2_w (after liposomal CDPC delivery);	$B_{0}$ = 11.7 T; $B_{1}$ = 2.7 µT (in vivo) T_sat_ = 3 s; Offsets: ±4 ppm (0.2 ppm steps)	Preclinical	[154]
Ischemic stroke	Amide at 3.5 ppm (↓) CIRI and ↑ Melatonin); Guanidium at 2.0 ppm (↓ CIRI and ↑ Melatonin)	↓ IL-1β; ↑ Arg1, CD206 (M2 polarization); H&E, TTC, TUNEL, and NeuN —reduced damage	$B_{0}$ = 7 T; $B_{1}$ = 1 µT Offsets: ±10 ppm; 51 offsets	Preclinical	[155]
SCI	Amide at 3.5 ppm (↑ Week 1); ↓ after riluzole	Iba-1 (↓ after riluzole), GFAP (no change); LFB (↑ after riluzole) & BBB score (↑ after riluzole)	$B_{0}$ $=\;9.4\;T;\;B_{1}$ = 1 μT T_sat_ = 2.0 s; Offsets: ±5 ppm; 33 offsets	Preclinical	[156]
Traumatic SCI	Amide at +3.5 ppm (↓); NOE at −1.6 ppm (↓); (Week 1 post-injury)	PET-TSPO (↑); Iba-1 and GFAP (↑)	$B_{0}$ = 9.4 T; CW T_sat_ = 2.0 s; Offsets: ±5 ppm;	Preclinical	[157]
Spinal Dorsal Nerve Root	Amide at 3.5 ppm (↑) NOE at −1.6 ppm (↓)	MRI (FA ↓, RD ↑, Pool Size Ratio ↓;	$B_{0}$ $=\;9.4\;T;\;B_{1}$ = 1.0 µT; CW T_sat_ = 5s; Offset: ±5 ppm (0.2 ppm steps)	Preclinical	[158]
HAND	Glu at 3 ppm (↓ in cortex, hippocampus, cortex at 12 WPI); Cr at 2 ppm (↓ in cortex and hippocampus at 6–12 WPI); NOE at −3.5 ppm (↑ in cortex and thalamus at 6 and 12 WPI)	IHC: p24, Iba-1, GFAP, HLA-DR activation (↑)	$B_{0}$ = 7 T; $B_{1}$ = 2 µT; T_sat_ = 2 s; Offsets: ±5 ppm 51 offsets (0.1–0.2 ppm steps)	Preclinical	[159]
HIV	Hydroxyl and amine of Lamivudine (3TC) and Emtricitabine (FTC) (↑)	Plasma & brain t 3TC/FTC via UPLC–MS/MS	$B_{0}$ $=\;7\;T;\;B_{1}$ = 2 μT; T_sat_ = 1 s; Offsets: ±5 ppm (0.2 ppm steps)	Preclinical	[160]
PD	Glu at 3.0ppm (↑)	Neuroinflammation inferred from astrocytic glutamate dysregulation	$B_{0}$ = 3 T; $B_{1}$ = 3 μT; Offsets: ±6 ppm 54 offsets	Clinical	[161]

Note ↑ increase; ↓ decrease; IHC: immunohistochemistry; p-tau: phosphorylated tau; MALDI: Matrix-Assisted Laser Desorption/Ionization; TEM: transmission electron microscopy; DEM: Demyelination; REM: Remyelination; MBP: Myelin Basic Protein; Cr: Creatine; Glu: Glutamate; DexCEST: Dextran-enhanced CEST; MI: Myo-inositol.

## Data Availability

Data sharing does not apply to this article as no new data were created or analyzed in this study.

## Abbreviations

The following abbreviations are used in this manuscript:

***Abbreviation***	**Full Form**
*^1^H-MRS*	Proton magnetic resonance spectroscopy
*AD*	Alzheimer’s disease
*AI*	Artificial intelligence
*ALS*	Amyotrophic lateral sclerosis
*AMO-CEST*	Attention-Based MultiOffset Deep Learning Reconstruction of Chemical Exchange Saturation Transfer
*APP/PS1*	Amyloid precursor protein/presenilin 1
*APTw*	Amide proton transfer-weighted
*AREX*	Apparent Exchange-dependent Relaxation
*ARV*	Antiretroviral
*ATP*	Adenosine triphosphate
*BacCEST*	Bacterial CEST
*BBB*	Blood–brain barrier
*BM3D*	Block matching combined with 3D filtering
*bSSFP*	Balanced steady-state free precession
*CD68**^+^*	Cluster of differentiation 68-positive
*CEST*	Chemical exchange saturation transfer
*CEST-VN*	Model-based Variational Network CEST
*CHI3L1*	Chitinase-3-like protein-1
*CIRI*	Cerebral ischemia–reperfusion injury
*CNNs*	Convolutional neural networks
*CNS*	Central nervous system
*Cr*	Creatine
*CrCEST*	Creatine CEST
*CryptoCEST*	Cryptococcus CEST
*CS*	Compressed sensing
*CVAE*	Conditional variational autoencoder
*CW*	Continuous-wave
*DAMPs*	Danger-associated molecular patterns
*DCE*	Dynamic contrast-enhanced
*DECENT*	Denoising CEST network
*DeepEMR*	Deep learning extrapolated semisolid magnetization transfer reference
*DeepCEST*	Deep neural network based CEST
*DexCEST*	Dextran-enhanced CEST
*DiaCEST*	Diamagnetic CEST
*dMRI*	Diffusion MRI
*DS*	Direct water saturation
*DTI*	Diffusion tensor imaging
*EAE*	Experimental autoimmune encephalomyelitis
*EMR*	Extrapolated magnetization transfer reference
*EndoCEST*	Endogenous CEST
*EPI*	Echo planar imaging
*FLAIR*	Fluid attenuated inversion recovery
*GABA*	Gamma-aminobutyric acid
*GAGs*	Glycosaminoglycans
*GagCEST*	Glycosaminoglycan CEST
*GFAP*	Glial fibrillary acidic protein
*Glu*	Glutamate
*GluCEST*	Glutamate-weighted CEST
*GlucoCEST*	Glucose CEST
*GlycoCEST*	Glycogen CEST
*GuanCEST*	guanidinium CEST
*GRASE*	Gradient and spin-echo
*GRE*	Gradient echo
*HAND*	HIV-associated neurocognitive disorder
*HD*	Huntington’s disease
*IDH*	Isocitrate dehydrogenase
*IHC*	Immunohistochemistry
*IL-1β*	Interleukin-1 beta
*IL-6*	Interleukin-6
*INRESP*	Implicit neural representation combined with explicit sparse prior
*k-Z PCA*	Z-spectrum principal component analysis
*ksw*	Exchange rate
*LD*	Lorentzian difference
*LPS*	Lipopolysaccharide
*LRAZ*	Low-rank approximation of the z-spectrum
*MALDI*	Matrix-assisted laser desorption/ionization
*MI*	Myo-inositol
*MRI*	Magnetic resonance imaging
*MRS*	Magnetic resonance spectroscopy
*MTC*	Magnetization transfer contrast
*MTC-MRF*	Magnetization transfer contrast MR fingerprinting
*MTRRex*	Magnetization transfer ratio with respect to exchange-dependent relaxation
*MTR_asym_*	Magnetization transfer ratio asymmetry
*NAA*	N-acetylaspartate
*NODDI*	Neurite orientation dispersion and density imaging
*NOE*	Nuclear Overhauser Effect
*ParaCEST*	Paramagnetic CEST
*PBCS*	Parallel blind compressed sensing
*pCr*	Phosphocreatine
*pCW*	Pulse-train
*PD*	Parkinson’s disease
*PET*	Positron emission tomography
*PTR*	Proton transfer ratio
*QSM*	Quantitative susceptibility mapping
*RF*	Radiofrequency
*rNOE*	Relayed Nuclear Overhauser Effect
*RPCA*	Robust principal component analysis
*SAE*	Sepsis-associated encephalopathy
*SCI*	Spinal cord injury
*SENSE*	Sensitivity encoding
*sLoFNet*	Single Lorentzian Fitting Network
*SNR*	Signal to noise ratio
*SPACE*	Sampling perfection with application-optimized contrasts using different flip angle evolution
*SPECT*	Single-photon emission computed tomography
*sTREM2*	Triggering Receptor Expressed on Myloid cells 2
*SVM*	Support vector machine
*SWI*	Susceptibility-weighted imaging
*TBI*	Traumatic Brain injury
*Cho*	Choline
*T_1_w*	T_1_-weighted
*T_2_w*	T_2_-weighted
*TNF-α*	Tumor necrosis factor-alpha
*T_sat_*	Saturation time
*T_ro_*	Readout time
*TSE*	Turbo spin echo
*TSPO*	Translocator protein
*UPDRS*	United Parkinson’s Disease Rating Scale
*WM*	White matter
